# 3D-QSAR, Docking, ADME/Tox studies on Flavone analogs reveal anticancer activity through Tankyrase inhibition

**DOI:** 10.1038/s41598-019-41984-7

**Published:** 2019-04-01

**Authors:** Sarfaraz Alam, Feroz Khan

**Affiliations:** 1 Metabolic & Structural Biology Department, CSIR-Central Institute of Medicinal and Aromatic Plants, P.O.-CIMAP, Lucknow, 226015 India; 2 Academy of Scientific and Innovative Research (AcSIR), Ghaziabad, 201002 India

## Abstract

Flavones are known as an inhibitor of tankyrase, a potential drug target of cancer. We here expedited the use of different computational approaches and presented a fast, easy, cost-effective and high throughput screening method to identify flavones analogs as potential tankyrase inhibitors. For this, we developed a field point based (3D-QSAR) quantitative structure-activity relationship model. The developed model showed acceptable predictive and descriptive capability as represented by standard statistical parameters r^2^ (0.89) and q^2^ (0.67). This model may help to explain SAR data and illustrated the key descriptors which were firmly related with the anticancer activity. Using the QSAR model a dataset of 8000 flavonoids were evaluated to classify the bioactivity, which resulted in the identification of 1480 compounds with the IC_50_ value of less than 5 µM. Further, these compounds were scrutinized through molecular docking and ADMET risk assessment. Total of 25 compounds identified which further analyzed for drug-likeness, oral bioavailability, synthetic accessibility, lead-likeness, and alerts for PAINS & Brenk. Besides, metabolites of screened compounds were also analyzed for pharmacokinetics compliance. Finally, compounds F2, F3, F8, F11, F13, F20, F21 and F25 with predicted activity (IC_50_) of 1.59, 1, 0.62, 0.79, 3.98, 0.79, 0.63 and 0.64, respectively were find as top hit leads. This study is offering the first example of a computationally-driven tool for prioritization and discovery of novel flavone scaffold for tankyrase receptor affinity with high therapeutic windows.

## Introduction

Tankyrase (TNKS) belongs to the diphtheria toxin-like ARTD (ADP-ribosyltransferase) enzyme superfamily (EC 2.4.2.30). They are also identified as poly (ADP-ribose) polymerases (PARPs)^1^. TNKS1 & TNKS2 are two isoforms of tankyrase and share imbricate functions and similar structures. This includes ANK (Ankyrin) repeat domain, SAM (sterile alpha molecule) domain, and catalytic PARP domain^2^. Altered tankyrase expression has been witnessed in different cancers, comprising fibro-sarcoma, glioblastoma, ovarian cancer, pancreatic adenocarcinoma, gastric cancer, breast cancers, bladder cancer, and colon cancer^3^. Their anticancer therapeutic perspective relates to the roles of tankyrases in telomere homeostasis and mitosis and Wnt signaling^4^. In normal cells, it found that telomeres get shorten in each cell division. This shortening signals cells to end cell division and regulates cellular senescence. In the case of cancer cells, the telomere length is maintained by the up-regulation of telomerase enzymes, which adds TTAGGG repeats to the end of chromosomes^5^. This enzyme regulates the lengths of telomeres indirectly through telomere repeat binding factor 1 (TRF1). The TRF1 protects telomeres from enzyme telomerase by binding with telomeric DNA. The ADP-ribosylation of TRF1 by TNKS1 inhibits the binding of TRF1 to telomeres, letting access to telomerase. Consequently, the partial knockdown of TNKS1 leads to telomere shortening^6^. Therefore, tankyrase inhibition offers a reasonable approach for cancer therapy, and as a result, the treatment of cancer cells in combination with telomerase and ARTD inhibitors leads to telomere shortening and cell death^7^. Tankyrase also required for correctness of structure and function of the mitotic spindle and centrosome, where the main protein substrate is NuMA^8^. The Abnormal role of Wnt signaling is complicated in many human cancers, and inhibitors of this system show anticancer activity *in vivo*^9^. The tankyrases find to control the action of the Wnt/β -catenin pathway by PARsylation and destabilization of the Axin proteins, stimulating its degradation by the ubiquitin-proteasome pathway. The inhibition of TNKS sustained the life of axin and surged the extent of the devastation complex of β-catenin, which led decrease levels of β-catenin and increased the levels of phosphorylated β–catenin triggering inhibition of the Wnt/β-catenin driven proliferation of cancer cells^10^. The prospective aspect of TNKS in disease-related cellular progressions have made them appealing drug targets. In the last decade, the tankyrase inhibitors have evidenced to be useful chemical probes and possible lead compounds, and therefore of great interest for the development of small molecule as tankyrase inhibitors. The consequence examples are, IWR-1 and IWR-2 which are tankyrase inhibitors and stabilize AXIN and inhibit WNT signaling and proliferation in APC-null DLD1 cancer cells^11^. In mice with inducible APC deficiency, exposure to JW55 reduce tumor load and decreases tumor area^12^. In 2010, Yashiroda *et al*. carried out a high-throughput screening of natural products library to restore growth inhibited by TANKs expression. Through this study, they identified flavone as a tankyrase inhibitor^13^. Flavones, belongs to the group of flavonoids, and have antioxidant properties and are present in a wide variety of food items. Flavones have also been shown to have antiproliferative properties in, prostate, lung, pancreas, colorectal, and ovarian cancer cells^14^.

This useful activity of flavone generates our interest in developing a tool for screening novel flavone derivatives/analogs that inhibit the tankyrase receptor. For this, the modern drug discovery aspects were applied such as 3D-QSAR (three-dimensional quantitative structure-activity relationship), molecular docking, ADMET (absorption, distribution, metabolism, excretion, toxicity), etc.^15,16^. The 3D-QSAR based on molecular interaction aptitudes can provide affluence information about the exact molecular characteristics essential for biological activity and served as a significant predictive tool, predominant in the design of pharmaceuticals^17^. The 3D-QSAR eradicates problems such as a restriction in the prediction of the stereochemistry of testing dataset and lack of recognition capability in search of active compound suffered by the classical 2D-QSAR studies^18^. In this paper, a 3D-QSAR were built and rigorously validated. The model gives information about a set of field points, which are associated with the activity of the compounds and analyze to find where the predicted activity values arise. Through this model, we here highlighted the structural features and revealed the key regulatory features governing the anticancer activity and predicting the tankyrase receptor affinity. With the advances in computer science and release of several compounds databases, there is a particular interest to filter these databases for finding any compound which can bind the desired receptors. This job may be conceded out by using the virtual screening methods, which helps the end user in filtering many compounds based on virtual model specifications. This model was employed for virtual screening of a large chemical library of flavone (~8000 compounds), resulting in 1480 top hits with an IC_50_ value of less than 5 µM. Furthermore, the molecular docking between 1480 flavonoids and tankyrase receptor performed to virtually screen top hits, and to identify the important substituents and mode of action of flavonoids. The top 200 compounds were then screened out by docking score and later analyzed for ADMET risk, which led to narrow down hits to 25 compounds with no risk. These compounds analyzed for drug-likeness, bioavailability and synthetic accessibility and alerts for PAINS & Brenk. Further, the screened compounds along with their metabolites were predicted and scrutinized for detailed pharmacokinetics (ADME) parameters. This led to identify eight active compounds, namely, F2, F3, F8, F11, F13, F20, F21 and F25 with predicted activity (IC_50_ value) of 1.59, 1, 0.62, 0.79, 3.98, 0.79, 0.63 & 0.64, respectively.

## Methods and Computational Details

### Parameters for QSAR model development, Data pool, and Structure preparation

The chemical structures of the training dataset active compounds selected from the prior reports/literature^19–27^. The two-dimensional (2D) chemical structures were drawn by using the ChemBioOffice Ultra 11.0 software (PerkinElmer/CambridgeSoft, UK). These structures were converted into three dimensional (3D) structures by utilizing the converter module of Forge v10 software (Cresset Inc., UK). For calculating the protonation state of the molecules the pH value is assuming as 7.0. The value of enzyme inhibition (experimental activity) expressed in (IC_50_) for training dataset and later transformed to its positive logarithmic scale by utilizing the formula: pIC_50_ = −log (IC_50_) and defined as a dependent variable.

### Conformation hunt, Pharmacophore generation, alignment, and built model calculations

The co-crystallized structure of drug target receptor complex was retrieved from RCSB Protein Data Bank (PDB) (https://www.rcsb.org/pdb) and split into protein receptor and reference ligand by using the software Forge v10 (Cresset Inc., UK). The target receptor used as a protein receptor excluding volume and the bound drug was used as a reference ligand to generate field pharmacophores and later, used as a bioactive reference conformation. This reference conformation further annotated with its calculated field points which derived in a three dimensional field point pattern. XED (eXtended Electron Distribution) force field was used to generate these field points. Through this, four diverse molecular fields such as positive and negative electrostatic, ‘shape’ (van der Waals), and ‘hydrophobic’ fields (a density function correlated with steric bulk and hydrophobicity) calculated. The field point’s pattern offers a condensed representation of the compound’s shape, hydrophobicity, and electrostatics. The reference conformer was then used to align the training and test set compounds by Maximum Common Substructure (MCS) and using customized thresholds^28^. The conformation hunt was done by very accurate and slow calculation method and the maximum number of conformations generated for each molecule set to 500. The RMSD (Root-mean-square deviation) cutoff set to 0.5 Å for atomic positions of duplicate conformers. Contrary, gradient cutoff for conformer minimization set to 0.1 kcal/mol. The XED force field were used to minimize all the conformers^29^. The energy window set to 3 kcal/mol. For building the 3D-QSAR model, the best matching low energy conformations to the template used. All the alignments were manually checked to ensure the best possible model. Hereafter, the initial training set of a total of 87 compounds divided into training and test-set by using the random selection method (Table S1 and S2). During QSAR modeling, the maximum number of components was fixed to 20, whereas the maximum distance for sample point set to 1.0 Å. The Y scrambles were set to 50, Volume fields, as well as Electrostatic properties used. The Forge v10 uses 50% Field similarity plus 50% Dice volume similarity. In the model building process, the partial least square (PLS) regression process was used through Forge’s field QSAR module, especially, the SIMPLS algorithm^30^.

### Validation of the QSAR model

The predictive ability of the derived 3D-QSAR model was confirmed by many statistical tests, which include correlation coefficient (r^2^), cross-validation regression coefficient (q^2^), in addition to similarity score (Sim). The (q^2^) were calculated by PRESS (prediction error sum of squares) and the SSY (sum of squares of deviation of the experimental values from their mean), as follows:

1 $${q}^{2}=1-\frac{press}{ssy}=1-\frac{{\sum }_{i=1}^{n}(Yexp-Ypred)2}{{\sum }_{i=1}^{n}(Yexp-Ymean)2}$$

Where Y_exp_ represents the experimental biological activity of the compound of the training set, however, the Y_pred_ represent the predicted activity of the compound of the training set, and Y_mean_ denotes the activity mean values of the training set compounds^31^. The robustness of the model was also validated through the determination of the coefficient in prediction, r^2^ test, using the following equation:

2 $${r}^{2}test=1-\frac{{\sum }_{i=1}^{n}(Ypredtest-Ytest)2}{{\sum }_{i=1}^{n}(Ytest-Ymean)2}$$

In the equation 2, the Y_predtest_ represent the predicted activity of the test set compound whereas the Y_test_ represents the experimental activity of test set compound, and Y_mean_ represents the mean values of the activity of training set compounds^31^. The developed model was calculated by the LOO (leave one out) method to optimize the activity model. Leave one out cross-validation (LOOCV) is considered to be the most effective approaches for validation of a model when there is a small training dataset. The training is carried out by using a data size of (N–1) and tested the remaining one. The N symbolizes the complete dataset. In the LOOCV methods, the training and testing compounds are repeated for an ‘N’ extent of time, so that to pass each data through the testing process^32^. The model has also been validated by using data, not in the training set.

### Visualization of SAR Activity Atlas models

The training dataset qualitatively visualized by the Bayesian approach. The Bayesian approach provided a proficient understanding of the hydrophobic, electrostatics, and shape features, which underlie the structure-activity relationship of a selected set of compounds. This valuable information attained by observing these models in three-dimensional form. The derived activity-atlas study shown the three diverse types of interrelated biochemical computed data, i.e., an average of actives, activity cliffs summary and regions explored analysis. The average of actives exhibited the common part in the active compounds. Whereas, the activity cliff summary specifies favorable & unfavorable hydrophobicity, positive & negative electrostatics sites, as well as the favorable shape of the active compounds. Simultaneously, regions explored exploration showed the areas of the aligned compounds which have been fully explored^32^.

### Generation of prediction set and field pattern contribution to the predicted activity

To select the best set of lead like compound, a field point-based virtual screening analysis accomplished. For this, a list of about 8000 small molecules retrieved from different databases and literature sources. Moreover, the retrieve compounds were screened through the developed 3D QSAR model for bioactivity prediction as well as by using the SAR field point’s compliances. The mismatched SAR field points of query/prediction set compounds removed.

### Molecular docking studies

#### Protein preparation

For protein preparation protocol, the three-dimensional crystallographic structures, and the coordinates of the target protein (Tankyrase 2, PDB ID: 4HKI) retrieved from the RCSB PDB database (https://www.rcsb.org/pdb). Initially, the protocol for protein preparation was to perform different tasks which includes inserting missing atoms in incomplete residues, deleting alternate conformations, modeling the missing loop regions, protonating titratable residues, predicted pKs (a negative logarithmic measure of the acid dissociation constant), and standardizing names of the atoms, and removed the heteroatoms or water molecules. The CHARMM force field employed for protein preparation^33^. Before processing, the hydrogen atoms were added^33^.

#### Protein-Ligand Docking

*In silico* docking simulations and post-docking visualization studies executed by using the software Discovery Studio v3.5 (Accelrys, USA, 2013)^34^. The docking exercise was completed by a LibDock program of Discovery Studio so that to reveal the bioactive binding site poses of potential inhibitors within the targets active site. The LibDock program used protein site features known as hot spots. These hot spots are of two types (polar & apolar). After this, the ligand poses placed into this polar and apolar receptor interactions site. In the parameterization step, the Merck Molecular Force Field (MMFF) force field used for energy minimization. For conformation generation, the CAESAR (Conformer Algorithm based on Energy Screening and Recursive build up) method used. All other docking and scoring parameters kept at their default sets. Additionally, to identify specific interacting residues of the receptor/target with a bound ligand, a 2D diagram of the docking stage was carried out. Further performed analysis for protein-ligand complexes and explain interactions between protein residues and bound ligands atoms, besides the binding site residues of the known receptor^35^.

### Bioavailability, drug-likeness and synthetic accessibility and ADMET screening

The Lipinski rule of five (Pfizer), Ghose (Amgen), Veber (GSK), Egan (Pharmacia) and Muegge (Bayer) rules were used for Drug likeness pre-screening studies. Bioavailability calculated by using the Abbott bioavailability score. Later, the studied compounds derived for PAINS, Brenk alerts, Lead likeness and also for synthetic accessibility scoring. To further validate and screen the query set (prediction set) compounds, the synthetic accessibility was measured using the SYLVIA-XT 1.4 module. This program offers a score on a scale from 1 to 10, where ‘1’ represents very easy to synthesize, and ‘10’ represent complex to synthesize. To measure score, the complexity of molecular structure, the complexity of the ring system, the number of stereo-centers, similar to commercially available compounds, and the potential for using critical synthetic reactions for each selected compound were independently weighted to render a single value for synthetic accessibility^36^. The pre-screening ADMET risk was calculated for each predicted active compounds in the query set so that to minimize the failure rate later due to the poor quantitative pharmacokinetics parameters compliance with standard anticancer drugs^37^.

### *In silico* pharmacokinetics, pharmacodynamics and toxicity studies

The different physicochemical properties were calculated for *in silico* evaluation of study compounds against standard pharmacokinetics parameters, such as Absorption, Distribution, Metabolism, Excretion (ADME) and later calculated their predicted toxicities by using ADMET Predictor^TM^ software (Simulations Plus Inc., USA). This study includes the quantitative measurement of drug-like properties such as, lipophilicity, solubility, pKa (negative logarithmic measure of acid dissociation constant), permeability, absorption, bioavailability, blood-brain barrier penetration, transporters, dermal and ocular penetration, plasma-protein binding, metabolism and drug-drug interaction, volume of distribution (V_d_), clearance, half-life, p-glycoprotein efflux and inhibition as well as inhibition of the hepatic organic anion transporting polypeptide (OATP-1B1) transporter, cytochromes P450 (CYP450) enzymes, and UDP-glucuronosyltransferases (UGTs). The MedChem Designer^TM^ software was used for metabolites prediction^38^. The safety of the compounds is an essential parameter for a successful drug. For this, the hepatotoxicity, neurotoxicity, androgen receptor toxicity, allergenic, mutagenicity, developmental toxicity were calculated along with the effect of compounds on some of the liver-associated enzymes such as alkaline phosphatase (ALP), gamma-glutamyltransferase (GGT), aspartate transaminase (AST), alanine transaminase (ALT), and lactate dehydrogenase (LDH) enzymes. This study led us to describe how the candidate compounds behave in the human body and also helpful to set dose-ranges^39^.

### Ethical approval

Appropriate guidelines and regulations were used to perform all the experiments.

## Results and Discussion

### Bioactive conformation hunt, Pharmacophore generation, and Compound alignment

**Figure 1:**
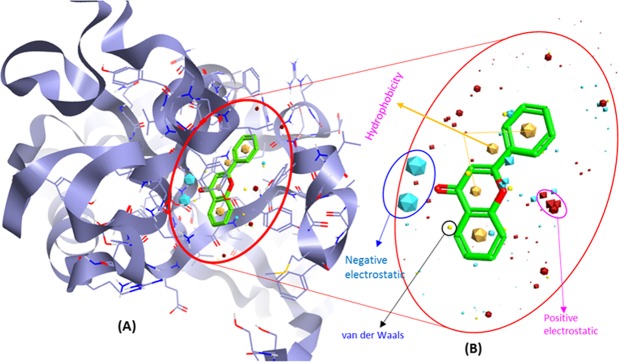
(**A**) Structural model of human tankyrase receptor 2 (PDB ID: 4HKI) used as protein excluded volume. (**B**) Representing the bioactive conformation of FLN (flavone) co-crystalized with tankyrase receptor 2 and used as a reference ligand to identify the three-dimensional field point’s pattern to generate field pharmacophores.

Prior studies showed the inhibition of tankyrase with flavone and its likely role in antiproliferative properties. Allowing for our interest in developing new flavone analogs that inhibit the tankyrase, a 3D-QSAR model for predicting the tankyrase receptor affinity has been built with the objective of providing a convenient tool for the identification, design, and optimization of new flavones ligands. For this, the protein-ligand x-ray crystal structure of tankyrase receptor 2 binds with FLN (Flavone) [PDB ID: 4HKI] was retrieved from the RCSB PDB database. Further, this structure is split into reference ligand, and protein, where 4HKI (Tankyrase) used as protein excluded volume (Fig. 1A) and FLN (Flavone) was used as reference ligand (Fig. 1B) to generate field pharmacophores.

**Figure 2:**
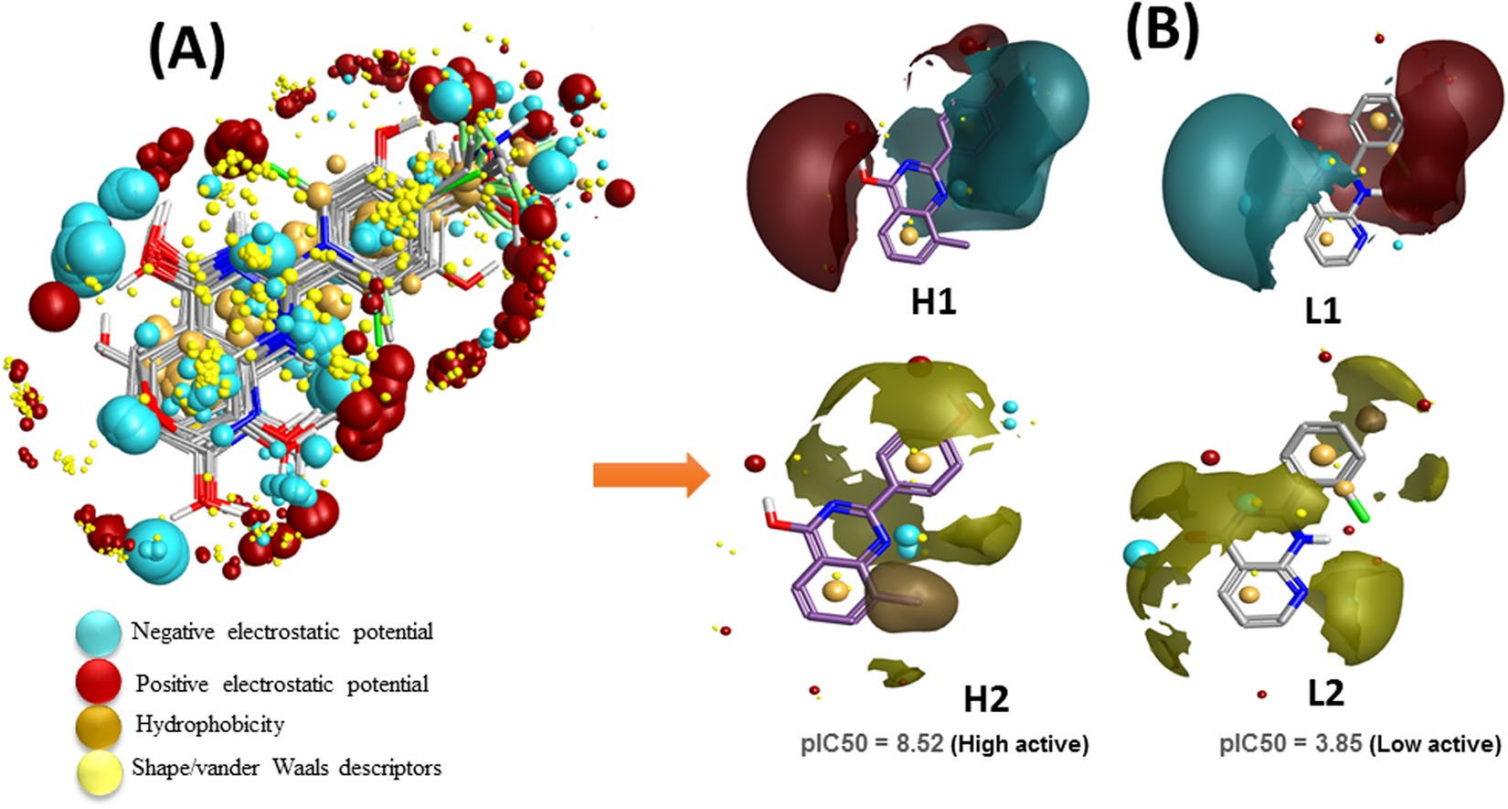
(**A**) Molecular representation of aligned training set compounds with their respective molecular field points. (**B**) Molecular representation of highly active training set compound (H1 & H2) and low active training set compound (L1 & L2) with their respective biological activity (pIC_50_). The cyan color shows negative field points, which indicates likely molecular regions interacting with positive or H-bond donors of the target protein, red color shows positive field points, which indicates likely molecular regions interacting with negative or H-bond acceptors of the target protein. Gold color shows hydrophobic field points, which indicates the regions with high polarizability or hydrophobicity, and yellow color shows Van der Waal field points.

The derived comprehension of bioactive conformation was further annotated through its calculated field points, leading to the identification of a 3D field point’s pattern. This feat the molecular field-based similarity technique for the search of conformation. This help to generate a pharmacophore template which bears a resemblance to the bioactive conformation, for virtual screening. The molecular depiction of aligned training set compounds with their respective molecular field points was provided in (Fig. 2A). In this figure, the negative field points are represented by the cyan color which specifies the molecular regions interacting with positive or H-bond donors of the target protein. On the other hand, the positive field points, which are represented by red color, indicates molecular regions interacting with H-bond acceptors/negative of the target protein. The gold color represents the hydrophobic field points, which specifies the regions with high polarizability or hydrophobicity whereas the yellow color displays van der Waal field points. Along with this the molecular depiction of highly active training set compound (H1 & H2; Fig. 2B) and low active training set compound (L1 & L2; Fig. 2B) with their corresponding biological activity (pIC_50_) were also provided. All the optimized 87 compounds then aligned to the selected pharmacophore template (reference conformation), which was later used to build the QSAR model.

### 3D QSAR model development and statistical analysis

**Figure 3:**
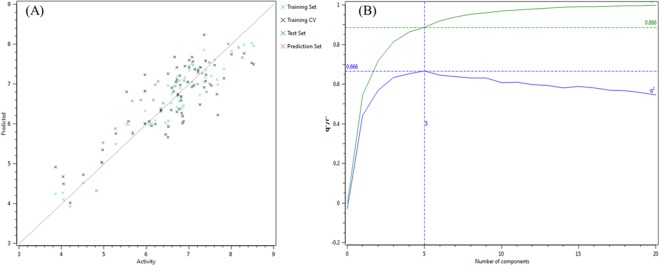
(**A**) Activity interactive graph plot between predicted and actual experimental activity. The graph plot shows separate data series for the training set (green color cross), test set (blue color cross), and training cross-validation set (black color cross). (**B**) 3D-QSAR model performance graph plot between cross-validation regression coefficient, q^2^ (blue color line), and the regression coefficient, r^2^ (green color line) ratio and the number of components.

For 3D-QSAR model development, the Field points based chemical descriptors used after the alignment of 87 compounds. For model development, the experimental biological activity (IC_50_) of the dataset changed to its positive logarithmic scale by applying the formula: pIC_50_ = −log (IC_50_) and describe it as a dependent variable. The software Forge uses a PLS regression protocol specifically exploit the SIMPLS algorithm. The dataset split into two subsets, i.e., 69 compounds were there in the training set (Table S1), and 18 compounds were there in the test set (Table S2) by using the random method. Finally, the 5-components model indicates good predictive and descriptive capabilities, as it was shown by the good regression coefficient (r^2^ = 0.89) and cross-validation regression coefficient (q^2^ = 0.67) values for the training and the cross-validated training set. Contrary, the test set showed a proper estimation and excellent cross-validated values of (r^2^ = 0.75) and presented in Table S3. The activity interactive graph exploration characterized the robustness of the developed QSAR model. The graph displays the comparison of experimental versus predicted activity plot along with cross-validation data point (Fig. 3).

### SAR mechanism of Flavones regulated by field points

#### Identification of field points (coefficient & variance) controlling anticancer activity

**Figure 4:**
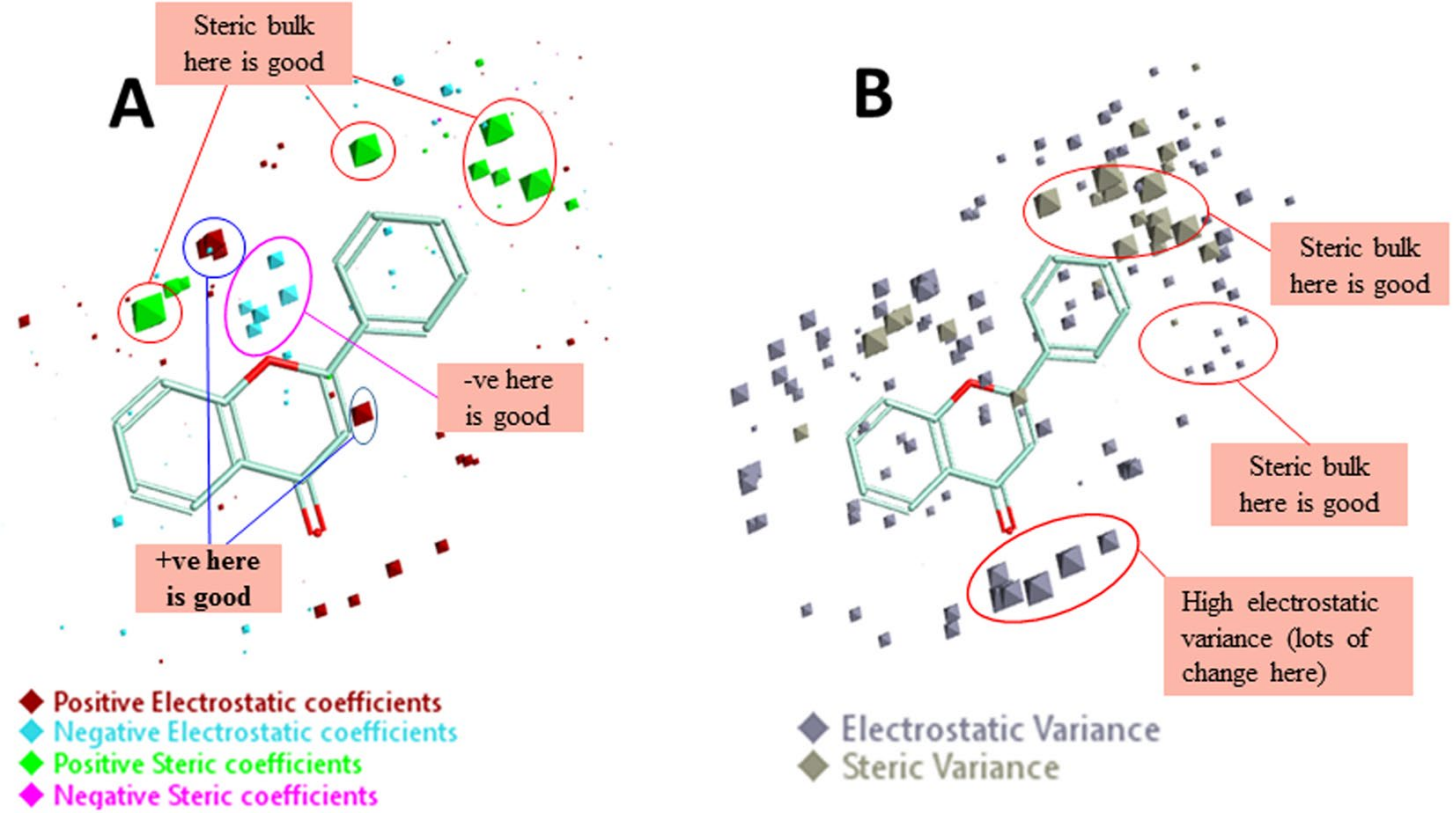
Molecular insight of flavone (reference molecule) representing the coefficients and variance field points modulating the bioactivity through the derived 3D-QSAR model. (**A**) Model coefficient field points in red color (positive electrostatics), cyan color (negative electrostatics) and green color (positive steric coefficient) show the region of a substantial effect on higher activity. (**B**) High electrostatic variance and high steric variance field points represent the region of high changes and points with low variance indicates the fields in that region with less or no changes.

To understand the SAR mechanism of flavone analogs, the QSAR model was envisioned in three-dimensional form. To achieve this the activity related field points, viz. coefficient and variance were explored for the training set compound is in the 3D structural form. The model displays the areas where the equation suggests that the local fields have a substantial impact on biological activity. The bigger the points, the stronger is the correlation between the electrostatic/steric fields in that position and hence higher affinity values. To apprehend the space field point’s localization, the QSAR model points were superposed to the structure of the reference compound. The high coefficient & variance field points were reflected truly significant correlating parameters in a robust model. The results of the structural analysis shown that the developed QSAR model was well dominated by the positive steric coefficient as specified by the large size of green color (Sterics+) and therefore, concluded that more steric bulk leads to higher activity (Fig. 4A). The other factors are positive (red color) and negative (cyan color) electrostatic coefficient. The electrostatic coefficient also plays a role in activity effects of substituents. The high variance (electrostatic & steric) field points signify the region of high changes whereas the points with low variance specifies the domain with less or no changes (Fig. 4B).

### SAR mechanism identification through Activity-Atlas visualization

The essential features of tankyrase receptor affinity for flavone analogs responsible for modulating the anticancer activity was revealed through SAR study and visualized by Activity Atlas. The Activity Atlas visualization method is a qualitative process and is valuable for the sum up the structure-activity data into three-dimensional maps which advise the designing and optimization of novel compounds. To achieve this, studies related to the average of actives and activity cliffs summary were studied in detail and discuss here. This study answers the following questions, what is common in active molecules and what is revealed by activity cliffs during SAR studies?

#### Results of Average of Actives analysis

**Figure 5:**
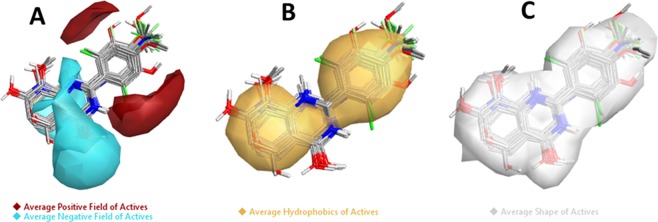
Molecular insight of SAR mechanism models, revealing the different lead optimization sites of active compounds including flavone, as detected through an average of actives analysis. (**A**) Active molecules, in general, have a positive field in this region (red color area), and active molecules, in general, have a negative field in this region (cyan color), (**B**) Active molecules in general make hydrophobic interactions in this region (yellow color), (**C**) Average shape of active molecules (white color).

The results in Fig. 5A represent the “Average Electrostatics of Actives” contributions show the regions where the active molecules, in general, show average positive field (red color area) and average negative field (cyan color area) whereas the “Average Hydrophobics of Actives” contributions show the regions where the active molecules, in general, make hydrophobic interactions with the receptor (Fig. 5B). The “Average Shape of actives” represented in Fig. 5C (white color) exhibit the average shape of active molecules. The identified fields along with shape and hydrophobic interactions associated with the high biological activity, and it implies that new molecules which show either positive or negative fields in the same region considered active.

#### Results of Activity cliff

**Figure 6:**
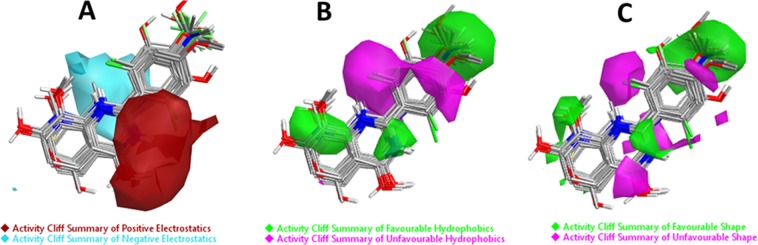
Molecular insight of SAR mechanism models, revealing the different lead optimization sites of active compounds including flavone, as detected through activity cliffs summary studies. (**A**) Positive electrostatics (red color) and negative electrostatics (cyan color), (**B**) Favorable hydrophobics (green color region) and unfavorable hydrophobics (magenta color region) and, (**C**) Favorable shape (green color region) and unfavorable shape (magenta color region).

The results of “Activity Cliff Summary of Electrostatics” analysis represented in Fig. 6A, which shows the molecular regions where comparison of all pairs of compounds revealed a more positive field (red color) and a more negative field (cyan color) increases anticancer activity. Whereas the “Activity Cliff Summary of Hydrophobics” shows regions where hydrophobic interaction is either beneficial (green regions) or detrimental (magenta regions) to biological activity (Fig. 6B). The “Activity Cliff Summary of Shape” is also calculated and results define the regions where steric bulk was either excellent (green color) or bad (magenta color). The green color represents the favorable shape, and thus in this region, more steric bulk leads to higher biological activity. On the other hand, the magenta color represents the unfavorable shape and illustrate that more steric bulk in this region leads to lower bioactivity (Fig. 6C).

#### Field contributions to predicted activity

**Figure 7:**
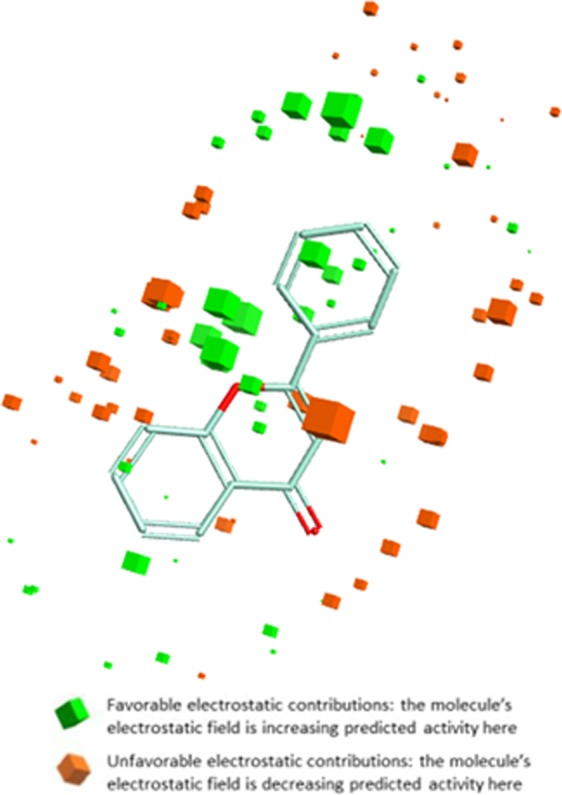
Molecular SAR mechanism of flavone analogs, representing different geometries of field contributions to the predicted activity. The green color represents favorable Electrostatic contributions whereas the orange color represents unfavorable Electrostatic contributions.

To assess how well flavone and its analogs fit on the developed field-based 3D-QSAR model, studies related to structural field point regions regulating predicted activity, and field contributions to predicted activity were completed. The results were displays with a green and orange color which showed these field points contributions to the predicted activity. Results showed that the green color (Electrostatics+) represent favorable electrostatic contributions and hence the molecule’s electrostatic field increasing predicted activity. Whereas the orange color (Electrostatics−) represent unfavorable electrostatic contributions, therefore, the molecule’s electrostatic field decreasing predicted activity (Fig. 7).

### Models validation through activity prediction of training and test set

Based on the developed structure-activity relationship models, the molecular features governing the anticancer activity of the active compounds mined for activity prediction of selected prediction (query) dataset compounds. Before that, the prediction performance was primarily calculated for training as well as test set compounds, by predicting antiproliferative/cytotoxic activity through the developed QSAR model and then matched the distance value (or error value). Also for comparison purpose, the distinct predicted activity including distance to model columns was assessed for each developed model. Later, required ligand fields were interpreted for target binding and detected molecular features were used in virtual screening.

### Ligand-based virtual screening for hits prediction

To propose a hit compound, a series of field-based 3D similarity experimentations were performed by using virtual screening approach. Subsequently, a set of 1480 compounds were identified by using the developed QSAR model descriptors. Among these, the compounds attending a value of ‘excellent’ were selected. It is suggested that the maximum of the features of these compound set was found similar to the training set and hence predicted activities could be expected to be reliable. Contrarily, compounds with ‘poor’ field point’s similarities were taken out to avoid unreliable or unpredictable activities shows by false positive compounds. Afterward, the anticancer activity prediction of top hit compounds completed by using the developed QSAR model. The developed QSAR model calculated the activity-dependent descriptors and then predicted the (IC_50_) of each compound and thus providing a potential inhibition range. The compounds with a predicted IC_50_ value of greater than 20 µM were removed. Further, the identified compounds were screened through Lipinski’s rule of five by accepting one rule violation and next through ADME parameters and toxicity risk for drug-likeness studies (Table S4).

### High binding affinity of Flavones on Tankyrase 2 revealed through Docking

**Figure 8:**
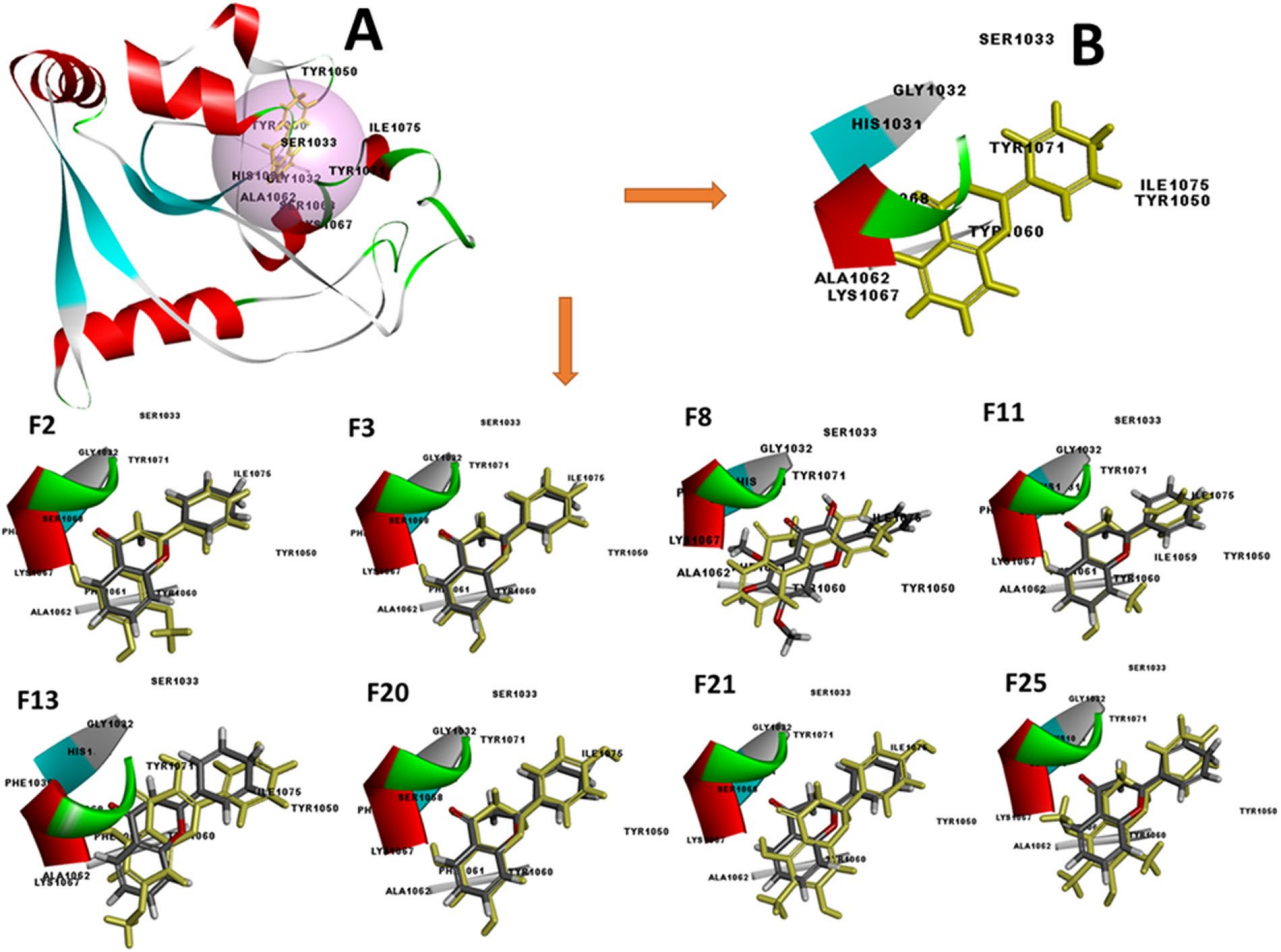
(**A**) Structural model of Tankyrase 2 (PDB: 4HKI) with ligand binding site (pink sphere). (**B**) Representing the conformation of standard compounds, namely, 4HKI-a. Representing the most active flavone derivatives, namely, F2, F3, F8, F11, F13, F20, F21, F22 and F25 along with superimposition of standard compound namely, 4HKI-a (yellow color).

The docking studies were carried out to virtual screen the 1400 compounds, screened in-prior through the derived 3D-QSAR model, as well as to identify the binding potency and poses of active molecules so that to reveal the molecular mechanism of action. Before docking studies, target protein (PDB: 4HKI) prepared. The compounds, when docked, demonstrated several poses, orientation and thus several configurations (Fig. 8). Each configuration characterized as a combined score of Vander Waals forces, hydrogen bonding, pi interaction as well as other relevant parameters, and signified in the form of a docking score namely, LibDock score (Table 1).

**Table 1:** Details of docking based parameters, namely, LibDock score, hydrogen bond pi interactions, and interactive amino acid residues of identified potential flavones analogs in the binding site pocket of target protein Tankyrase 2 (PDB ID: 4HKI).

Sr. No.	Compound	LibDock Score	H-Bond	Pi-Interactions	Interactive amino acid residues
1.	**F2**	134.16	GLY_1032_ SER_1068_	TYR_1071_ (3) HIS_1031_ LYS_1067_	PHE_1030_, HIS_1031_, GLY_1032_, SER_1033_, TYR_1050_, TYR_1060_, PHE_1061_, ALA_1062_, LYS_1067_, SER_1068_, TYR_1071_, ILE_1075_
2.	**F3**	106.42	GLY_1032_ SER_1068_	TYR_1071_ (2) HIS_1031_ (2) LYS_1067_	PHE_1030_, HIS_1031_, GLY_1032_, SER_1033_, TYR_1050_, TYR_1060_, PHE_1061_, ALA_1062_, LYS_1067_, SER_1068_, TYR_1071_, ILE_1075_
3.	**F8**	127.58	GLY_1032_ (2)	HIS_1031_ (2) TYR_1071_ (2)	PHE_1030_, HIS_1031_, GLY_1032_, SER_1033_, TYR_1050_, TYR_1060_, PHE_1061_, ALA_1062_, LYS_1067_, SER_1068_, TYR_1071_, ILE_1075_
4.	**F11**	133.65	GLY_1032_ SER_1068_	TYR_1071_ LYS_1067_	PHE_1030_, HIS_1031_, GLY_1032_, SER_1033_, TYR_1050_, ILE_1059_, TYR_1060_, PHE_1061_, ALA_1062_, LYS_1067_, SER_1068_, TYR_1071_, ILE_1075_
5.	**F13**	133.97	GLY_1032_ (2) SER_1068_	TYR_1050_ TYR_1071_ (2) HIS_1031_	PHE_1030_, HIS_1031_, GLY_1032_, SER_1033_, TYR_1050_, TYR_1060_, PHE_1061_, ALA_1062_, LYS_1067_, SER_1068_, TYR_1071_, ILE_1075_
6.	**F20**	136.09	GLY_1032_ SER_1068_	TYR_1071_ (2) HIS_1031_ LYS_1067_	PHE_1030_, HIS_1031_, GLY_1032_, SER_1033_, TYR_1050_, TYR_1060_, PHE_1061_, ALA_1062_, LYS_1067_, SER_1068_, TYR_1071_, ILE_1075_
7.	**F21**	138.41	None	TYR_1071_ (2) HIS_1031_ LYS_1067_	HIS_1031_, GLY_1032_, SER_1033,_ TYR_1050_, TYR_1060_, ALA1062, LYS_1067,_ SER_1068_, TYR_1071_, ILE_1075_
8.	**F25**	124.57	None	TYR_1071_ LYS_1067_	HIS_1031_, GLY_1032_, SER_1033_, TYR_1050_, TYR_1060_, PHE_1061_, ALA_1062_, LYS_1067_, SER_1068_, TYR_1071_
9.	**4HKI-a** **(Standard)**	121.02	GLY_1032_	LYS_1067_ TYR_1071_	HIS_1031_, GLY_1032_, SER_1033_, TYR_1050_, TYR_1060_, ALA_1062_, LYS_1067_, SER_1068_, TYR_1071_, ILE_1075_

The higher LibDock score indicates a higher chance of ligand-protein binding. The docked compounds, namely, F2, F3, F8, F11, F13, F20, F21, F25 and standard compound gives a LibDock score of 134.16, 106.42, 127.58, 133.65, 133.97, 136.09, 138.41, 124.57 and 121.02, respectively. All the compounds were found to make pi-interactions, whereas, except F21 and F25, all the compounds identified showed hydrogen bond formation. Results indicate that the candidate compounds showed a good docking score in comparison to standard compound, thus indicate high binding affinity of these hit compounds. The detail docking score with hydrogen bond, pi interactions along with interactive amino acid was summarized in Table 1. Additionally, a 2D diagram was provided in Fig. S1 to reveal the different molecular interactions. These interactions denoted by separate colors, e.g., the electrostatic interaction denoted by pink color and purple specifies a covalent bond, whereas the green color depicted Van der Waals molecular interactions. Solvent accessibility of the ligand atoms and the amino acid residues displayed by blue shading where high shading implies more exposure to the solvent. The results indicate that the compounds were able to bind well within the binding site pocket of tankyrase 2 and showed almost a similar binding pattern (Fig. 8). These results provided a molecular level understanding to infer that identified compounds are promiscuous and might be a potential inhibitor of tankyrase 2, and may bind well at the active site.

### Compliance with a standard range of drug-likeness, bioavailability, synthetic accessibility and alerts for PAINS & Brenk filters

Drug-likeness studies qualitatively measure the chance of a molecule to turn into an oral drug concerning its bioavailability. Five different rules-based filters were used to calculate the drug & lead likeness for 25 query set compounds (prediction set). The results exhibited that all the compounds show good drug-likeness score with zero violation of understudy drug-likeness rules. All the compounds showed a lead-likeness with zero violation of the standard range, except compound F22 which showed the XLogP 3 > 3.5. Apart from this, the PAINS and Brenk method used for identification of potentially problematic fragments which yields false-positive biological output and so the results of this screening study indicate that compounds F2, F3, F8, F11, F13, F20, F21, F22, and F25 did not show any such fragment. Rest of the compound show violations, due to the inclusion of fragment namely, catechol and hydroquinone in the chemical structure of query dataset compounds (Table 2).

**Table 2:** Details of different drug-likeness rules, bioavailability, lead-likeness, synthetic accessibility, and alerts for PAINS & Brenk.

Compound	Drug-likeness Rules						Alerts		Lead likeness	Synthetic Accessibility
	Lipinski (Pfizer)	Ghose (Amgen)	Veber (GSK)	Egan (Pharmacia)	Muegge (Bayer)	Bioavailability score	PAINS	Brenk		
F1	0	Yes	Yes	Yes	Yes	0.55	Catechol_A*	Catechol**	Yes	3
F2	0	Yes	Yes	Yes	Yes	0.55	0	0	Yes	3.15
F3	0	Yes	Yes	Yes	Yes	0.55	0	0	Yes	2.96
F4	0	Yes	Yes	Yes	Yes	0.55	Catechol_A*	Catechol**	Yes	3.04
F5	0	Yes	Yes	Yes	Yes	0.55	Catechol_A*	Catechol** Hydroquinone^#^	Yes	3.08
F6	0	Yes	Yes	Yes	Yes	0.55	0	Hydroquinone^#^	Yes	3.17
F7	0	Yes	Yes	Yes	Yes	0.55	Catechol_A*	Catechol**	Yes	3.05
F8	0	Yes	Yes	Yes	Yes	0.55	0	0	Yes	3.37
F9	0	Yes	Yes	Yes	Yes	0.55	0	Hydroquinone^#^	Yes	3.15
F10	0	Yes	Yes	Yes	Yes	0.55	0	Hydroquinone^#^	Yes	3.24
F11	0	Yes	Yes	Yes	Yes	0.55	0	0	Yes	3.11
F12	0	Yes	Yes	Yes	Yes	0.55	0	Hydroquinone^#^	Yes	3.25
F13	0	Yes	Yes	Yes	Yes	0.55	0	0	Yes	3.18
F14	0	Yes	Yes	Yes	Yes	0.55	Catechol_A*	Catechol**	Yes	3.37
F15	0	Yes	Yes	Yes	Yes	0.55	Catechol_A*	Catechol**	Yes	3.12
F16	0	Yes	Yes	Yes	Yes	0.55	Catechol_A*	Catechol**	Yes	3.02
F17	0	Yes	Yes	Yes	Yes	0.55	Catechol_A*	Catechol**	Yes	3.10
F18	0	Yes	Yes	Yes	Yes	0.55	Catechol_A*	Catechol**	Yes	3.12
F19	0	Yes	Yes	Yes	Yes	0.55	0	Hydroquinone^#^	Yes	3.34
F20	0	Yes	Yes	Yes	Yes	0.55	0	0	Yes	3.01
F21	0	Yes	Yes	Yes	Yes	0.55	0	0	Yes	3.13
F22	0	Yes	Yes	Yes	Yes	0.55	0	0	XLOGP3 >3.5	3.35
F23	0	Yes	Yes	Yes	Yes	0.55	Catechol_A*	Catechol**	Yes	3.14
F24	0	Yes	Yes	Yes	Yes	0.55	Catechol_A*	Catechol**	Yes	3.19
F25	0	Yes	Yes	Yes	Yes	0.55	0	0	Yes	3.34

*

**

#.

Beside this, a rule-based method for lead likeness calculated for the studied candidate compounds, and violated descriptors identified (Table 2). Herewith the query compounds also screened for synthetic accessibility appraisal. To quantify, the complexity of the molecular structure & ring system, number of stereocenters, and the potential for using critical synthetic reactions were individualistically weighted to compromise a particular value for synthetic accessibility. The compounds with high scores or tough to synthesize were removed. The results showed that the score for the compounds was in the range of 3.12–4.27, in comparison to doxorubicin, which gives a score of 5.81. The obtained results revealed that the compounds could be synthesized easily. The Abbot Bioavailability score predicts the chance of compound to have at least 10% oral bioavailability in rat or quantifiable Caco-2 cell line permeability experiment and defined by a probability score of 11%, 17%, 56%, and 85%. The candidate compounds showed a score of 56%, indicating good bioavailability.

### *In silico* pharmacokinetics compliance evaluation

The completion of a drug’s journey over the body measured in terms of ADMET (absorption, distribution, metabolism, elimination, and toxicity. The results for these ADMET parameters were achieved for studied compounds, i.e., F2, F3, F8, F11, F13, F20, F21, and F25, by calculating the diverse physicochemical and biopharmaceutical features. The solubility features were assessed for different stages, especially native water solubility, fasted state intestinal fluid, fed state intestinal fluid and fasted state gastric fluid, and delivered in the quantitative term. The results indicated that the native water solubility was 0.014, 0.107, 0.091, 0.109, 0.02, 0.151, 0.147, and 0.029 mg/mL for lead compounds, namely, F2, F3, F8, F11, F13, F20, F21 and F25. The solubility measurement of the compounds in intestinal fluid at fasting state was 0.181, 0.158, 0.185, 0.139, 0.121, 0.346, 0.376 and 0.041 mg/mL, while the solubility in intestinal fluid at fed state was 0.339, 0.444, 0.256, 0.392, 0.247, 0.468, 0.475 and 0.165 mg/mL, whereas the solubility in gastric fluid at fasted state was 0.08, 0.072, 0.046, 0.054, 0.039, 0.119, 0.094, and 0.038 mg/mL for F2, F3, F8, F11, F13, F20, F21 and F25 respectively. The MLogP (Moriguchi model of octanol-water partition coefficient) of the compound was identified as 1.55, 2.24, 1.02, 2.49, 1.55, 1.45, 1.70 and 2.46, whereas the octanol-water partition coefficient was 2.86, 3.09, 2.40, 3.32, 2.9, 2.42, 2.67, and 3.51 while the octanol-water distribution coefficient was 2.67, 2.78, 2.38, 3.03, 2.82, 2.10, 2.28 and 3.46 for top hit compounds *viz*., F2, F3, F8, F11, F13, F20, F21 and F25, respectively. The molecular diffusion coefficient in water was identified as 0.83, 0.87, 0.78, 0.83, 0.83, 0.86, 0.82 and 0.76 cm^2^/s X 10^5^, whereas the logarithm of air-water partition coefficient was −10.37, −10.86, −10.56, −10.57, −11.00, −12.06, −11.81 and −8.71 atm*m^3^/mol for top hit compounds *viz*., F2, F3, F8, F11, F13, F20, F21 and F25, respectively. The degree of ionization (pKa) has considerable effect on solubility and permeability was also calculated and resulted as 11.52, 9.48, 9.82, 9.67, 9.70, 9.81, 9.75, and 8.3 for top hit compounds *viz*., F2, F3, F8, F11, F13, F20, F21 and F25 respectively (Table S5). Lipophilicity is the compound’s ability to dissolve into the lipophilic (non-aqueous) medium and correlated to various models of drug properties affecting ADMET that includes permeability, absorption, solubility, metabolism, distribution, plasma protein binding, elimination, and toxicity. Results revealed that all compounds show an optimal range of LogP, which describe a good balance of permeability and solubility existence and thus shows good oral bioavailability. The LogD value of compounds F2, F3, F8, F11, F13, F20, and F21 was predicted to be 2.67, 2.78, 2.38, 3.03, 2.82, 2.10, and 2.28, respectively. All the compounds, except F25, indicate an ideal range and compounds generally showed favorable intestinal absorption, thus expressive a good balance of solubility and permeability but the metabolism process may be minimized, owing to lesser binding to metabolic enzymes. The compound F25 shows a LogD value of 3.46 indicates that compounds have favorable permeability; however, absorption was lower, remaining to lower solubility. The metabolism may increase in this range, thus increased the binding potential to metabolic enzymes. The results predicted for the volume of distribution (V_d_) was 0.58, 0.45, 1.05, 0.5, 1.4, 0.38, 0.37, and 1.35 L/kg for top hit compounds *viz*., F2, F3, F8, F11, F13, F20, F21 and F25, respectively. Results indicate that the compound has a small volume of distribution and hence, mainly distributed in the extracellular fluid.

Drug compounds encounter numerous diverse membrane barriers such as hepatocyte membrane, gastrointestinal epithelial cells, blood capillary wall, glomerulus, restrictive organ barriers (e.g., Blood-Brain Barrier), and the target cell. These permeability predictions can help to understand the ADMET results and the cell-based bioassays. Results indicated that the permeability over human skin was predicted to be 11.53, 14.20, 5.26, 11.92, 10.44, 5.05, 4.02 and 8.87 cm/s × 10^7^ for compounds *viz*., F2, F3, F8, F11, F13, F20, F21, and F25, respectively. The MDCK COS (MadinDarby canine kidney, cells-on-sheet) permeability was 581.73, 338.72, 699.35, 480.63, 600.54, 137.34, 189.57 and 811.91 cm/s × 10^7^ for compounds F2, F3, F8, F11, F13, F20, F21 and F25 respectively. The prediction for human jejunal effective permeability (Peff) was 5.17, 4.94, 5.06, 5.33, 5.56, 2.99, 3.22 and 4.78 cm/s × 10^4^ for compounds F2, F3, F8, F11, F13, F20, F21 and F25, respectively. Likewise, the permeability through the rabbit cornea was identified as 151.4, 123.50, 102.9, 130.61, 154.71, 59.78, 65.81 and 138.93 cm/s × 10^7^ for compounds F2, F3, F8, F11, F13, F20, F21 and F25. The molecular diffusion coefficient in water, which affect the solubility and permeability were predicted to be 0.83, 0.87, 0.78, 0.83, 0.83, 0.86, 0.82 and 0.76 cm^2^/s × 10^5^ for compounds F2, F3, F8, F11, F13, F20, F21 and F25, respectively. The compounds showed a low probability, to cross the BBB. The percent unbound to blood plasma proteins was 3.16, 2.83, 7.1, 2.31, 4.33, 4.51, 3.21 and 3.59 for compounds F2, F3, F8, F11, F13, F20, F21 and F25, respectively. This result indicates that there would be less chance of compound retention in drug plasma compartment without reducing the V_d_. It also implies that the metabolism and clearance rate will not decrease, and there would be no prolongation of half-life (t_1/2_). The blood to plasma concentration ratio was anticipated to be 0.53, 1.08, 0.54, 0.87, 0.73, 1.06, 0.85, and 0.87 for compounds F2, F3, F8, F11, F13, F20, F21 and F25, respectively. Results indicate that there would be less possibility of compound binding with erythrocytes and thus may perhaps not exceed the hepatic blood flow. The result for the fraction unbound in human liver microsomes was identify to be 0.62, 0.57, 0.70, 0.51, 0.59, 0.74, 0.7 and 0.4 for compounds F2, F3, F8, F11, F13, F20, F21 and F25, respectively.

Metabolism plays an important role in the bioavailability of drugs as well as drug-drug interactions. Only the free drug can bind with drug-metabolizing enzymes. The cytochrome P450 enzymes (CYPs) might be the most significant class of enzyme to study the metabolic behavior of lead compounds. This study might help to understand the mechanism of drug disposition, efficacy, and toxicity. To achieve this, the hit compounds evaluated for either substrate or inhibitors of CYPs along with CYPs of Human Liver Microsomes (HLM). Mostly all the compounds were found to be a substrate of CYP1A2, except F25, whereas, for CYP2C8, only compound F2 was found to be the substrate. Additionally, the compounds F2, F3, F11, F20, and F21 were found to be the substrate of CYP2C9. Moreover, only compound F13 was found to be the substrate of CYP2C19 and compound F25 was found to be the substrate of CYP3A4. In identifying the affinity of studies compound with CYP-P450 enzymes in quantitative terms, the Michaelis-Menten constant (K_m_), maximum metabolic rate (V_max_) and intrinsic clearance (C_Lint_) calculated, which provide the knowledge of the rate of metabolism. Results revealed that for predicting the site of enzyme CYP1A2, the K_m_ value was 28.53, 33.79, 18.55, 16.59, 7.42, 158.31 and 49.91 μM, whereas the V_max_ constant was 1.76, 4.22, 5.34, 1.18, 1.99, 5.19 and 8.04 nM/min/nM and the C_Lint_ was 3.21, 6.49, 14.96, 3.69, 13.92, 1.70, and 8.37 μL/min/mg for compounds F2, F3, F8, F11, F13, F20, and F21, respectively. Likewise for predicting the site of enzyme CYP2C9, the K_m_ value was 3.43, 5.38, 3.81, 3.59 and 37.40 μM, whereas the V_max_ constant was 0.38, 0.35, 0.24, 0.49 and 4.95 nM/min/nM and the C_Lint_ was 8.14, 4.8, 4.61, 9.97 and 9.67 μL/min/mg for compounds F2, F3, F11, F20 and F21, respectively. Additionally, that for predicting the site of enzyme CYP2C19, the K_m_ value was 61.866 μM, whereas the V_max_ constant was 2.35 nM/min/nM and the C_Lint_ was 0.53 μL/min/mg for compound F13. On the other hand for predicting the site of enzyme CYP3A4 mediated metabolism the K_m_ value was 46.37 μM and the HLM Km value was 1055.87 μM, whereas the V_max_ constant was 6.36 nM/min/nM and HLM V_max_ constant was 1.25 nM/min/nM, and the C_Lint_ was 15.23 μL/min/mg, and the HLM C_Lint_ was 1.19 for compound F25. The overall calculated intrinsic clearance in human liver microsomes was 19.5, 12.63, 17.30, 13.97, 17.58, 7.72, 7.3 and 20.34 μL/min/mg for compounds F2, F3, F8, F11, F13, F20, F21, and F25, respectively. However, results of inhibition studies showed that all the compound might inhibit CYP1A2, CYP2C9, and CYP2D6, except compound F2 which did not inhibit the CYP2D6. Whereas no compounds were found to inhibit the CYP2C19. Moreover, the compounds when studies for CYP3A4, it was observed that compound F2, F8 and F13 may inhibit the CYP3A4. Results showed that all compounds might inhibit the CYP3A4 mediated midazolam and testosterone metabolism. The calculated K_i_ value for midazolam based inhibition was 63.13, 14.75, 28.07, 14.3, 28.42, 14.89, 14.64, and 16.87 for compounds F2, F3, F8, F11, F13, F20, F21, and F25, respectively.

**Figure 9:**
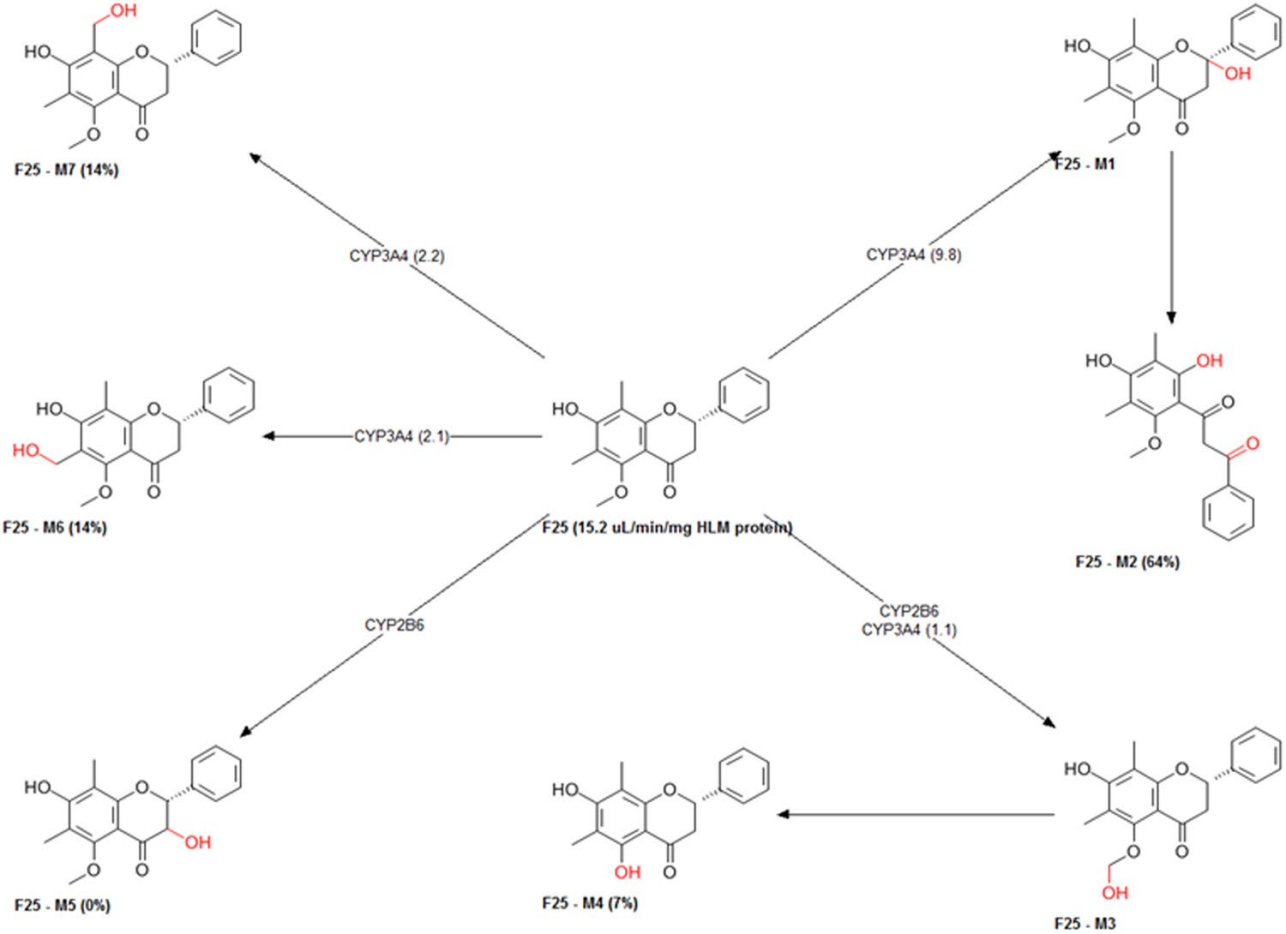
The predictive metabolites and sites of metabolism by CYPs for candidate compound F25.

The metabolism of candidate compounds produces numerous metabolites and these metabolites may have diverse pharmacological and physicochemical properties. These metabolism properties were explored *in silico* and summarized by predicting the metabolic sites as well as metabolites, and type of CYPs involved (Figs 9 and S2(a–h)). Furthermore to understand the mechanism of xenobiotic elimination, studied compounds namely, F2, F3, F8, F11, F13, F20, F21, and F25 screened for activity on UGT (Uridine 5′-diphosphate-glucuronosyltransferases family) enzymes, which catalyzes xenobiotics/drugs in phase II metabolism and transform the small molecules to water-soluble form, which may lead to the easy elimination of xenobiotics. Results showed that compound F2 might act as a substrate of UGT1A1, 1A3, 1A8, 1A9, 1A10, 2B7, and 2B15. Whereas the compound F25 may serve as a substrate of UGT1A1, 1A3, 1A4, 1A9, 1A10 and 2B15. On the other hand, the compound F3, F8, F11, F13, F20, F21 may act as a substrate of UGT1A1, 1A3, 1A8, 1A9, 1A10, and 2B15. These results imply that all these compounds may be eliminated more easily from the body. However, results indicate that studied compounds may not act as a substrate of PgP. Thus there may not be any chance to reduce the efficacy of the drug. The compounds inhibited the OATP1B and, therefore there might be a chance of drug-drug interaction with these compounds (Table S6).

### Predicted toxicology of identified leads (flavone analogs)

The identified flavones F2, F3, F8, F11, F13, F20, F21, and F25 studied in detail for *in silico* toxicity. Results showed that the maximum recommended therapeutic dose (MRTD) was above 3.16 mg/kg/day for all compounds. Results showed no sign of hERG (human Ether-a-go-go-Related Gene) inhibition. Thus, there might be no adverse cardiac effect. Results did not show any drug-induced phospholipidosis (intracellular accretion of phospholipids) which linked with undesirable clinical side effects, e.g., QT prolongation, myopathy, hepatotoxicity, nephrotoxicity, or pulmonary dysfunction. The possible reproductive toxicity calculated for compound and results showed no sign of such toxicity, except compound F25. Drug-induced hepatotoxicity, which roots the acute and chronic liver disease consequences in elevated levels of AST, ALT, ALP, and LDH enzymes. Candidate compounds tested for the elevation of these enzymes. Results showed that except compound F3, which may elevate the level of ALT, rest all compounds become normal for such enzymes. On the other hand, the GGT enzyme was elevated by compound F2 and F13, whereas F8 and F13 elevated the LDH enzyme (Table 3). Additionally, the compound when studying for androgen receptor toxicity, it was found that all the compounds were non-toxic, except compound F11 and F25, whereas in case of estrogen receptor toxicity in rats, compound F8 and F25 were safe and showed no such toxicity (Table 3).

**Table 3:** Details of calculated toxicity risk parameters for long-term or high doses use of predicted active top hit flavone analogs.

Identifier		F2	F3	F8	F11	F13	F20	F21	F25
**Maximum recommended therapeutic dose administered as an oral dose (mg/kg/day)**		Above_ 3.16	Above_ 3.16	Above_ 3.16	Above_ 3.16	Above_ 3.16	Above_ 3.16	Above_ 3.16	Above_ 3.16
**Estrogen receptor (rats)**		Toxic	Toxic	Nontoxic	Toxic	Toxic	Toxic	Toxic	Nontoxic
**Estro_RBA**		0.0004	0.002	Nontoxic	0.002	0.006	0.004	0.006	Nontoxic
**Androgen receptor toxicity**		Nontoxic	Nontoxic	Nontoxic	Toxic	Nontoxic	Nontoxic	Nontoxic	Toxic
**Andro_RBA**		Nontoxic	Nontoxic	Nontoxic	0.01	Nontoxic	Nontoxic	Nontoxic	0.02
**Allergenic skin sensitization (mice)**		None	None	None	None	None	None	None	None
**Allergenic respiratory sensitization in rat**		None	Sensitizer	None	None	None	Sensitizer	None	None
**Fathead minnow lethal toxicity after 96 h of exposure (mg/L)**		4.21	6.10	1.03	2.99	1.08	9.42	4.83	0.93
**Tetrahymena pyriformis growth inhibition toxicity (mmol/L)**		1.27	1.08	1.37	1.05	1.46	0.94	1.08	1.2
***Daphnia magna*** **(water flea) lethal toxicity after 48 h of exposure (mg/L)**		7.41	29.61	2.26	14.18	0.93	65.03	31.21	2.57
**Bio concentration factor**		26.00	14.53	13.19	20.59	20.08	7.80	8.68	57.4
**Biodegradation**		No	No	No	No	No	No	No	No
**Likelihood of the hERG potassium channel inhibition in human**		No	No	No	No	No	No	No	No
**Affinity towards hERG K+ channel and potential for cardiac toxicity (mol/L)**		4.44	4.68	3.94	4.52	4.09	4.51	4.38	4.44
**LD50 for lethal rat acute toxicity (mg/kg)**		520.75	576.12	1066.41	496.48	774.12	773.10	616.99	861.1
**Tumorogenic dose rat (mg/kg/day)**		351.19	487.76	261.06	421.83	310.1	617.42	559.65	178.53
**Tumorogenic dose mice (mg/kg/day)**		527.06	7236.21	747.69	3858.85	896.22	9444.9	5646.46	2065.66
**Triggering the mutagenic chromosomal aberrations**		Toxic	Toxic	Toxic	Toxic	Toxic	Toxic	Toxic	Nontoxic
**Causing phospholipidosis**		Nontoxic	Nontoxic	Nontoxic	Nontoxic	Nontoxic	Nontoxic	Nontoxic	Nontoxic
**Reproductive/developmental toxicity**		Nontoxic	Nontoxic	Nontoxic	Nontoxic	Nontoxic	Nontoxic	Nontoxic	Toxic
**Hepatotoxicity**	Levels of ALP enzyme	Normal	Normal	Normal	Normal	Normal	Normal	Normal	Normal
	Levels of GGT enzyme	Elevated	Normal	Normal	Normal	Elevated	Normal	Normal	Normal
	Levels of LDH enzyme	Normal	Normal	Elevated	Normal	Elevated	Normal	Normal	Normal
	Levels of AST enzyme	Normal	Normal	Normal	Normal	Normal	Normal	Normal	Normal
	Levels of ALT enzyme	Normal	Elevated	Normal	Normal	Normal	Normal	Normal	Normal
**Mutagenicity (pure compound)**	TA97 and/or TA1537 strains of *S. typhimurium*	Positive	Negative	Negative	Negative	Negative	Negative	Negative	Negative
	TA98 strain of *S. typhimurium*	Negative	Negative	Negative	Negative	Negative	Negative	Negative	Negative
	TA100 strain of *S. typhimurium*	Negative	Negative	Negative	Negative	Negative	Negative	Negative	Negative
	*S. typhimurium* and/or WP2 uvrA strain of *E. coli*	Negative	Negative	Negative	Negative	Negative	Negative	Negative	Negative
	TA1535 strain of *S. typhimurium*	Negative	Negative	Negative	Negative	Negative	Negative	Negative	Negative
**Mutagenicity (microsomal rat liver metabolites)**	TA97 and/or TA1537 strains of *S. typhimurium*	Positive	Negative	Positive	Negative	Positive	Negative	Negative	Negative
	TA98 strain of *S. typhimurium*	Negative	Negative	Negative	Negative	Negative	Negative	Negative	Negative
	TA100 strain of *S. typhimurium*	Negative	Negative	Negative	Negative	Negative	Negative	Negative	Positive
	TA102 strain of *S. typhimurium*	Negative	Negative	Negative	Negative	Negative	Negative	Negative	Negative
	TA1535 strain of *S. typhimurium*	Negative	Negative	Negative	Negative	Negative	Negative	Negative	Negative

Results showed that studied compounds might not reduce sperm concentration. All the compounds showed non-allergenic skin sensitization. On the other hand two compounds, i.e., F3 and F20 were found to cause allergenic respiratory sensitization. An alternative to animal testing was used to predict the dose-dependent toxicities such as LD_50_ and tumorigenic dose (TD) values. Whereas the water flea (*Daphnia magna*) lethal toxicity afterward 48 hours of exposure was calculated to be 7.41, 29.61, 2.26, 14.18, 0.93, 65.03, 31.21 and 2.57 mg/L for F2, F3, F8, F11, F13, F20, F21, and F25, respectively. The results suggest that 351.19 and 527.06 mg/kg/day of compound F2, 487.76 and 7236.21 mg/kg/day of F3, 261.06 and 747.69 mg/kg/day of F8, 421.83 and 3858.85 mg/kg/day of F11, 310.1 and 896.22 mg/kg/day of F13, 617.42 and 9444.9 mg/kg/day of F20, 559.65 and 5646.46 mg/kg/day of F21, 178.53 and 2065.66 mg/kg/day of F25 were required to induce tumorigenesis in the rat and mice. The identified compounds along with their metabolites were measured for mutagenicity by using the Ames test on a different strain of *Salmonella typhimurium*. The results suggest that all the studied compounds were non-mutagenic in pure form, except compound F2, which shows mutagenicity for the TA97 strain of *S. typhimurium*. In the case of metabolites, the results indicate that all the compounds were non-mutagenic for TA98, TA100, TA102 and TA1535 strain of *S. typhimurium*. On the other hand, the compound F2, F8, and F13 show a little chance of mutagenicity for the TA97 strain of *S. typhimurium*, if administered for long-term or in high dosage form (Table 3).

## Conclusion

The studied work deals with the development of a field-based 3D QSAR model on flavone series of natural small molecules for exploring the mechanism of inhibition on Tankyrase. The studied mechanism of action unravel the underlying structure-activity relationship and therefore, may speed up the designing as well as the identification of the novel, potent and selective flavone ligands targeting tankyrase. The structural studies and chemical space analysis made it promising to evaluate which class of flavones can inhibit the Tankyrases. The studies also offer potential insights for the region where the active molecules lie and also signify the average shape of active molecules. It also represents the areas where the positive and negative charges of active molecules lie, as well as the hydrophobic regions. By using this method, the user can, in particular, inspect how the model predicted compounds and make a supposition regarding the possible changes which make a molecule to fit the model and the changes required for the specific position to increase its biological activity. The ADMET study here given helps in optimizing the compounds regarding its pharmacological effect. These results could offer a significant boost to the consciousness of full perspective of virtual screening for the identification of hits compounds with more potent biological activity and negligible or no toxicity. This generated work may pave the way for selection of compound as well as designing of new chemical scaffolds or novel combinatorial libraries of analogs/derivatives.

## Supplementary information

Supplementary information

## Data Availability

All data generated or analyzed in the study included in Supplementary Information files.
