# Supplementary material for: 3D-QSAR, Docking, ADME/Tox studies on Flavone analogs reveal anticancer activity through Tankyrase inhibition
Source: Sci Rep. 2019 Apr 1;9:5414. doi: 10.1038/s41598-019-41984-7 (PMC6443786; doi:10.1038/s41598-019-41984-7)
Supplement: Supplementary file 1 — Supplementary information [file 41598_2019_41984_MOESM1_ESM.pdf]

3D-QSAR, Docking, ADME/Tox studies on Flavone analogs reveal anticancer activity through Tankyrase inhibition

Sarfaraz Alam[1, 2] and Feroz Khan*[1, 2]

[1]Metabolic & Structural Biology Department, CSIR-Central Institute of Medicinal and Aromatic Plants, P.O.CIMAP, Lucknow, 226015, India
[2]Academy of Scientific and Innovative Research (AcSIR), Ghaziabad- 201002, India

*Correspondence to be addressed:
Department of Metabolic & Structural Biology,
CSIR-Central Institute of Medicinal and Aromatic Plants
PO-CIMAP, Kukrail Picnic Spot Road, Lucknow-226015, Uttar Pradesh, India
Tel.: +91 522 271 7668, Fax: +91 522 234 2666
E-mail: f.khan@cimap.res.in

Table S1: Comparison of experimental and predicted activities of training data set molecules based on QSAR model.

Sr. No.	Title	Structure (SMILE)	Experimental Activity* (µM)	Predicted activity*(µM)	Distance to model
**1.**	**FL-1**	O=C1C=C(Oc2ccccc12)c3ccccc3	6.85	6.2	Excellent
**2.**	**FL-4**	Oc1cccc(C2=CC(=O)c3ccccc3O2)c1	6.5	6	Excellent
**3.**	**FL-6**	Clc1ccccc1[C@H]2NC(=O)c3ccccc3N2	4.51	4.5	Excellent
**4.**	**FL-7**	Oc1ccc(C2=CC(=O)c3ccccc3O2)cc1O	6.77	6.6	Excellent
**5.**	**FL-8**	O=C1c2ccccc2N[C@H](N1)c3ccc(cc3)C	5.57	6	Excellent
**6.**	**FL-9**	O=C1c2cccnc2N[C@H](N1)c3ccccc3C	4.03	4.3	Excellent
**7.**	**FL-12**	O=C1c2ccccc2N[C@H](N1)c3ccccc3C	4.04	4.1	Excellent
**8.**	**FL-13**	O=C1C=C(Oc2cc(O)ccc12)c3cccc(O)c3	6.2	6.1	Excellent
**9.**	**FL-14**	O=C1C=C(Oc2ccccc12)c3ccc(C(C)C)cc3	7.14	7.4	Excellent
**10.**	**FL-15**	Clc1ccc(C2=Nc3ccccc3C(=O)N2)cc1	7.27	7.4	Excellent
**11.**	**FL-16**	Clc1ccccc1[C@@H]2NC(=O)c3cccnc3N2	3.85	4.3	Excellent
**12.**	**FL-17**	Clc1ccc([C@@H]2NC(=O)c3cccnc3N2)cc1	6.12	6	Excellent
**13.**	**FL-18**	O=C1C=2COCCC2N=C(N1)c3cscc3	6.87	6.5	Excellent
**14.**	**FL-19**	O=C1c2cccnc2N[C@@H](N1)c3ccc(cc3)C	5.27	5.5	Excellent
**15.**	**FL-20**	Brc1ccc(C2=Nc3ccccc3C(=O)N2)cc1	7	7.4	Excellent
**16.**	**FL-21**	Fc1ccccc1[C@H]2NC(=O)c3cccnc3N2	4.2	3.9	Excellent
**17.**	**FL-23**	Oc1ccc(C2=CC(=O)c3ccc(O)cc3O2)cc1O	6.06	6	Excellent
**18.**	**FL-25**	O=C1C=C(Oc2ccccc12)c3ccc([N+]([O-])=O)cc3	6.85	6.4	Excellent
**19.**	**FL-27**	Oc1cc(O)cc2c1C(=O)C=C(O2)c3ccc(O)cc3	5.53	6	Excellent
**20.**	**FL-29**	Oc1ccc2C(=O)C=C(Oc2c1)c3ccc(OC)cc3	6.34	6.3	Excellent
**21.**	**FL-30**	Oc1cccc2C(=O)NC(=Nc12)c3ccc(C)cc3	7.36	7.1	Excellent
**22.**	**FL-31**	Oc1cc(O)cc2c1C(=O)C=C(O2)c3ccc(O)c(O)c3	5.96	6.3	Excellent
**23.**	**FL-33**	Clc1ccc(C=2NC(=O)C=3COCCC3N2)cc1	6.83	6.4	Excellent
**24.**	**FL-34**	O=C1c2cccnc2N[C@H](N1)c3ccc(OC)cc3	5.67	5.8	Excellent
**25.**	**FL-36**	Brc1ccc(C=2NC(=O)C=3COCCC3N2)cc1	7.09	7.5	Excellent
**26.**	**FL-37**	O=C1C2=C(N=C(N3CCN(CC3)C)N1)CCCC2	6.52	6.1	Excellent
**27.**	**FL-38**	Brc1ccc(C2=Nc3c(O)cccc3C(=O)N2)cc1	7.82	7.4	Excellent
**28.**	**FL-40**	O=C1c2cccnc2N[C@H](N1)c3ccc(C(C)C)cc3	6.45	6	Excellent
**29.**	**FL-41**	FC(F)(F)c1ccc(C=2NC(=O)C3=C(N2)CCCC3)cc1	6.79	6.9	Excellent
**30.**	**FL-42**	O=C1c2ccccc2N[C@@H](N1)c3ccc(C(C)(C)C)cc3	6.82	6.6	Excellent
**31.**	**FL-45**	Fc1ccc(C2=Nc3c(O)cccc3C(=O)N2)cc1	6.58	6.5	Excellent
**32.**	**FL-46**	O=C1C=2COCCC2N=C(N1)c3ccc(OC)cc3	7.07	7.1	Excellent
**33.**	**FL-48**	Clc1cc(Cl)ccc1[C@H]2NC(=O)c3cccnc3N2C	4.96	5	Excellent
**34.**	**FL-51**	Brc1ccc(C=2NC(=O)c3cccc(OC)c3N2)cc1	6.97	7	Excellent
**35.**	**FL-52**	Clc1ccc(C=2NC(=O)c3cccc(OC)c3N2)cc1	7.21	7.4	Excellent
**36.**	**FL-54**	Fc1ccc(C=2NC(=O)c3cccc(OC)c3N2)cc1	6.34	6.4	Excellent
**37.**	**FL-55**	FC(F)(F)Oc1ccc(C=2NC(=O)C=3COCCC3N2)cc1	7.26	6.7	Excellent

**38.**	**FL-56**	O=C1C=2COCCC2N=C(N1)c3ccc(cc3)C(OC)=O	6.69	7	Excellent
**39.**	**FL-57**	Oc1cccc2c(O)nc(nc12)-c3ccc(N)cc3	6.66	6.9	Excellent
**40.**	**FL-59**	Oc1c2cccc(c2nc(n1)-c3ccccc3)C	7.39	7.2	Excellent
**41.**	**FL-60**	Oc1cccc2c(O)nc(nc12)-c3ccccc3	6.74	6.7	Excellent
**42.**	**FL-61**	FC(F)(F)c1ccc(cc1)C=2NC(=O)C=3COCCC3N2	7.12	7	Excellent
**43.**	**FL-63**	Oc1c2cccc(O)c2nc(n1)-c3ccc(O)cc3	7.07	7.5	Excellent
**44.**	**FL-64**	O=C1C=2COCCC2N=C(N1)c3ccc(N(C)C)cc3	7.39	7.6	Excellent
**45.**	**FL-65**	Fc1ccc(-c2nc(O)c3ccccc3n2)cc1	5.96	6.8	Excellent
**46.**	**FL-67**	O=C1c2cccc(c2N=C(N1)c3ccc(CN)cc3)C	7	7.5	Excellent
**47.**	**FL-69**	O=C1C=2COCCC2N=C(N1)c3ccc(C(O)(C)C)cc3	7.66	7.1	Excellent
**48.**	**FL-70**	FC(F)(F)c1ccc(-c2cc3c(O)cccc3c(O)n2)cc1	7.6	7.6	Excellent
**49.**	**FL-73**	Oc1c2cccc(c2nc(n1)-c3ccc(O)cc3)C	8.52	8	Excellent
**50.**	**FL-74**	FC(F)(F)c1ccc(cc1)-c2nc(O)c3CSCCc3n2	8.3	8	Excellent
**51.**	**FL-75**	FC(F)(F)c1ccc(C=2NC(=O)[C@@H]3CSC[C@H]3N2)cc1	6.9	7.2	Excellent
**52.**	**FL-77**	Oc1cccc2c(O)nc(nc12)-c3ccc([N+]([O-])=O)cc3	6.72	7.2	Excellent
**53.**	**FL-90**	Fc1cccc(-c2ccc3C(C)=CC(=O)Nc3c2)c1	6.82	6.7	Excellent
**54.**	**FL-94**	FC(F)(F)c1ccc(-c2nc(O)c3cccc(c3n2)C)cc1	8	7.8	Excellent
**55.**	**FL-98**	Fc1ccc(-c2ccc3C(C)=CC(=O)Nc3c2)cc1	6.51	7	Excellent
**56.**	**FL-99**	Oc1c2cccc(c2nc(n1)-c3ccc([N+]([O-])=O)cc3)C	7.52	7.2	Excellent
**57.**	**FL-109**	O=C1c2cccc(c2N=C(N1)c3ccc(CNC(OCc4ccccc4)=O)cc3)C	7.36	7.9	Excellent
**58.**	**FL-112**	Clc1cc(ccc1-c2ccc3C(C)=CC(=O)Nc3c2)C(O)=O	7.68	7	Excellent
**59.**	**FL-116**	O=C1C=C(Oc2ccccc12)c3ccc(C)cc3	6.72	7.1	Excellent
**60.**	**FL-117**	Fc1ccc(C2=CC(=O)c3ccccc3O2)cc1	5.96	6	Excellent
**61.**	**FL-118**	O=C1c2cccnc2N[C@H](N1)c3ccccc3	4.94	5	Excellent
**62.**	**FL-120**	Oc1ccc(C2=Nc3ccccc3C(=O)N2)cc1	6.72	6.4	Excellent
**63.**	**FL-121**	Fc1ccc(C2=Nc3c(cccc3C(=O)N2)C)cc1	7.4	7.3	Excellent
**64.**	**FL-122**	O=C1C=2COCCC2N=C(N1)c3ccc(cc3)C	7.21	7.4	Excellent
**65.**	**FL-123**	O=C1c2ccccc2N=C(N1)c3ccc([N+]([O-])=O)cc3	6.59	6.8	Excellent
**66.**	**FL-124**	O=C1c2ccccc2N[C@H](N1)c3ccc(C(C)C)cc3	6.64	6.9	Excellent
**67.**	**FL-126**	O=C1c2cccc(OC)c2N=C(N1)c3ccc(C)cc3	6.78	7.1	Excellent
**68.**	**FL-130**	Oc1c2cccc(c2nc(n1)-c3ccc(N)cc3)C	8.48	8	Excellent
**69.**	**FL-136**	O=C1C=C(c2ccc(cc2N1)-c3ccccc3)C	6.74	6.7	Excellent

*Experimental and predictive value is in pIC_{50}.

Table S2: Comparison of experimental and predicted activities of test data set molecules based on QSAR model.

**Sr. No.**	**Title**	**Structure (SMILE)**	**Experimental Activity** (µM)	**Predicted activity** (µM)	**Distance to model**
**1.**	**FL-2**	O=C1c2ccccc2N[C@@H](N1)c3ccccc3	5.26	5.9	Excellent
**2.**	**FL-3**	Fc1ccccc1[C@H]2NC(=O)c3ccccc3N2	4.82	4.3	Excellent
**3.**	**FL-5**	O=C1c2ccccc2N=C(c3ccc(cc3)C)C1	6.27	7.1	Excellent
**4.**	**FL-10**	O=C1c2cccnc2C=C(N1)c3ccccc3	4.97	5.5	Excellent
**5.**	**FL-11**	Clc1ccc([C@H]2NC(=O)c3ccccc3N2)cc1	6.39	6.5	Excellent
**6.**	**FL-22**	O=C1c2ccccc2N=C(N1)c3ccc(N)cc3	6.16	6.6	Excellent
**7.**	**FL-26**	O=C1c2cccc(c2N=C(N1)c3ccc(C)cc3)C	8.17	7.9	Excellent
**8.**	**FL-32**	Clc1ccc(C2=Nc3c(cccc3C(=O)N2)C)cc1	7.48	7.5	Excellent
**9.**	**FL-35**	FC(F)(F)c1ccc(C2=Nc3ccccc3C(=O)N2)cc1	6.88	7.1	Excellent
**10.**	**FL-39**	Brc1ccc(C2=Nc3c(cccc3C(=O)N2)C)cc1	8.26	7.7	Excellent
**11.**	**FL-43**	Oc1c2ccccc2nc(n1)-c3ccccc3	5.66	6.6	Excellent
**12.**	**FL-47**	Clc1ccc(C2=Nc3c(O)cccc3C(=O)N2)cc1	7.31	7.3	Excellent
**13.**	**FL-68**	O=S(=O)(c1ccc(cc1)C=2NC(=O)C=3COCCC3N2)C	7.2	7	Good
**14.**	**FL-71**	Oc1c2ccccc2nc(n1)-c3ccc(OC)cc3	6.21	7	Excellent
**15.**	**FL-92**	Oc1c2cccc(c2nc(n1)-c3ccc(OC)cc3)C	7.92	8	Excellent
**16.**	**FL-103**	Clc1cc(N)ccc1-c2ccc3C(C)=CC(=O)Nc3c2	7.41	6.8	Excellent
**17.**	**FL-125**	FC(F)(F)c1ccc(C2=NC3=C(SCCC3)C(=O)N2)cc1	7.49	7	Excellent
**18.**	**FL-143**	O=C1C=C(c2ccc(cc2N1)-c3ccccc3OC)C	5.86	6.6	Excellent

*Experimental and predictive value is in pIC_{50}.

Table S3: 3D-QSAR model statistics.

Component	R^2	Q^2	Test R^2
**Component 1**	0.542	0.441	0.16
**Component 2**	0.718	0.57	0.519
**Component 3**	0.814	0.633	0.623
**Component 4**	0.862	0.653	0.678
**Component 5**	0.886	0.666	0.75
**Component 6**	0.919	0.645	0.732
**Component 7**	0.938	0.638	0.773
**Component 8**	0.953	0.631	0.798
**Component 9**	0.96	0.631	0.796
**Component 10**	0.968	0.606	0.771
**Component 11**	0.974	0.61	0.761
**Component 12**	0.98	0.597	0.781
**Component 13**	0.984	0.594	0.764
**Component 14**	0.988	0.582	0.747
**Component 15**	0.99	0.587	0.724
**Component 16**	0.992	0.58	0.702
**Component 17**	0.994	0.57	0.675
**Component 18**	0.995	0.566	0.677
**Component 19**	0.996	0.558	0.679
**Component 20**	0.997	0.546	0.689

Table S4: ADMET Risk screening of query set compounds.

Sr. No.	Compound Name	ADMET_ Risk	Absn_ Risk	RuleOf5	CYP_ Risk	MUT Risk	MUT Code	TOX Risk
**1.**	**F1**	0	0	0	0	1	m1	0
**2.**	**F2**	0	0	0	0	1	S1	0
**3.**	**F3**	0	0	0	0	0	Nil	0
**4.**	**F4**	0	0	0	0	1	S1	0
**5.**	**F5**	0	0	0	0	1	S1	0
**6.**	**F6**	0	0	0	0	1	m1	0
**7.**	**F7**	0	0	0	0	1	S1	0
**8.**	**F8**	0	0	0	0	1	m1	0
**9.**	**F9**	0	0	0	0	1	S1	0
**10.**	**F10**	0	0	0	0	1	m1	0
**11.**	**F11**	0	0	0	0	0	Nil	0
**12.**	**F12**	0	0	0	0	1	m1	0
**13.**	**F13**	0	0	0	0	1	m1	0
**14.**	**F14**	0	0	0	0	1	m1	0
**15.**	**F15**	0	0	0	0	0	Nil	0
**16.**	**F16**	0	0	0	0	1	S1	0
**17.**	**F17**	0	0	0	0	1	m1	0
**18.**	**F18**	0	0	0	0	1	m1	0
**19.**	**F19**	0	0	0	0	1	m1	0
**20.**	**F20**	0	0	0	0	0	Nil	0
**21.**	**F21**	0	0	0	0	0	Nil	0
**22.**	**F22**	0	0	0	0	1	m1	0
**23.**	**F23**	0	0	0	0	1	m1	0
**24.**	**F24**	0	0	0	0	1	m1	0

**25.**	**F25**	0	0	0	0	0	Nil	0

Table S5: Bio-physicochemical properties of the candidate compound.

Identifier	F2	F3	F8	F11	F13	F20	F21	F25
**S+Acidic_pKa**	11.52	9.48	9.82	9.67	9.70	9.81	9.75	8.3
**DiffCoef**	0.83	0.87	0.78	0.83	0.83	0.86	0.82	0.76
**MlogP**	1.55	2.24	1.02	2.49	1.55	1.45	1.70	2.46
**S+logP**	2.86	3.09	2.40	3.32	2.9	2.42	2.67	3.51
**S+logD**	2.67	2.78	2.38	3.03	2.82	2.10	2.28	3.46
**logHLC**	-10.37	-10.86	-10.56	-10.57	-11.00	-12.06	-11.81	-8.71
**S+Peff**	5.17	4.94	5.06	5.33	5.56	2.99	3.22	4.78
**S+MDCK**	581.73	338.72	699.35	480.63	600.54	137.34	189.57	811.91
**Perm_Cornea**	151.4	123.50	102.9	130.61	154.71	59.78	65.81	138.93
**Perm_Skin**	11.53	14.20	5.26	11.92	10.44	5.05	4.02	8.87
**S+Sw**	0.01	0.11	0.09	0.11	0.02	0.15	0.15	0.03
**S+pH_Satd**	5.98	5.39	6.09	5.41	6.11	5.32	5.27	6.15
**S+S_Intrins**	0.01	0.11	0.09	0.11	0.02	0.15	0.15	0.03
**SolFactor**	751.45	217.63	268.55	222.10	611.53	184.3	192.47	507.45
**S+S_pH**	0.02	0.21	0.1	0.21	0.02	0.31	0.36	0.03
**S+FaSSGF**	0.08	0.07	0.05	0.05	0.04	0.12	0.09	0.04
**S+FaSSIF**	0.18	0.16	0.19	0.14	0.12	0.35	0.38	0.04
**S+FeSSIF**	0.34	0.44	0.26	0.39	0.25	0.47	0.48	0.17
**SupSatn**	SupSat	SupSat	SupSat	SupSat	SupSat	SupSat	SupSat	SupSat
**BBB_Filter**	Low	Low	Low	Low	Low	Low	Low	Low
**LogBB**	-0.39	-0.29	-0.47	-0.35	-0.49	-0.39	-0.52	-0.21
**PrUnbnd**	3.16	2.83	7.1	2.31	4.33	4.51	3.21	3.59
**Vd**	0.58	0.45	1.05	0.5	1.4	0.38	0.37	1.35
**RBP**	0.53	1.08	0.54	0.87	0.73	1.06	0.85	0.87
**S+fumic**	0.62	0.57	0.70	0.51	0.59	0.74	0.7	0.4
**Pgp_Substr**	No	No	No	No	No	No	No	No
**Pgp_Inh**	Yes	No	No	No	No	No	No	No
**OATP1B1_Inh**	No	No	No	No	No	No	No	No
**HIVI-ST**	4.37	4.03	4.97	4.16	4.65	4.09	4.32	4.17
**HIVI-TC**	4.32	3.75	4.46	3.81	4.27	3.98	3.99	3.81
**Absn_Risk**	0	0	0	0	0	0	0	0
**RuleOf5**	0	0	0	0	0	0	0	0

Table S6: Metabolism study of the candidate compound.

Identifier	F2	F3	F8	F11	F13	F20	F21	F25
**CYP1A2_Inh**	Yes	Yes	Yes	Yes	Yes	Yes	Yes	Yes
**CYP1A2_Substr**	Yes	Yes	Yes	Yes	Yes	Yes	Yes	No
**CYP1A2_Sites**	C14(933); C13(767); C15(767)	C2(920); C6(560)	C23(944); C21(648); C15(585)	C2(862)	C21(970); C2(840); C15(628)	C2(917)	C15(388); C13(388); C6(236); C8(226)	Non-Substrate
**CYP1A2_Km**	28.53	33.79	18.55	16.59	7.42	158.31	49.91	Non-Substrate
**CYP1A2_Vmax**	1.76	4.22	5.34	1.18	1.99	5.19	8.04	Non-Substrate
**CYP1A2_CLint**	3.21	6.49	14.96	3.69	13.92	1.70	8.37	Non-Substrate
**CYP2A6_Substr**	No	No	No	No	No	No	No	No
**CYP2A6_Sites**	Non-Substrate	Non-Substrate	Non-Substrate	Non-Substrate	Non-Substrate	Non-Substrate	Non-Substrate	Non-Substrate
**CYP2B6_Substr**	No	No	No	No	No	No	No	Yes
**CYP2B6_Sites**	Non-Substrate	Non-Substrate	Non-Substrate	Non-Substrate	Non-Substrate	Non-Substrate	Non-Substrate	C22(799); C8(731)
**CYP2C8_Substr**	Yes	No	No	No	No	No	No	No

**CYP2C8_Sites**	C21(921)	Non-Substrate	Non-Substrate	Non-Substrate	Non-Substrate	Non-Substrate	Non-Substrate	Non-Substrate
**CYP2C9_Inh**	Yes	Yes	Yes	Yes	Yes	Yes	Yes	Yes
**CYP2C9_Substr**	Yes	Yes	No	Yes	No	Yes	Yes	No
**CYP2C9_Sites**	C14(889); C21(872)	C2(851)	Non-Substrate	C2(800)	Non-Substrate	C2(871)	C21(655)	Non-Substrate
**CYP2C9_Km**	3.43	5.38	Non-Substrate	3.81	Non-Substrate	3.59	37.40	Non-Substrate
**CYP2C9_Vmax**	0.38	0.35	Non-Substrate	0.24	Non-Substrate	0.49	4.95	Non-Substrate
**CYP2C9_CLint**	8.14	4.8	Non-Substrate	4.61	Non-Substrate	9.97	9.67	Non-Substrate
**CYP2C19_Inh**	No	No	No	No	No	No	No	No
**CYP2C19_Substr**	No	No	No	No	Yes	No	No	No
**CYP2C19_Sites**	Non-Substrate	Non-Substrate	Non-Substrate	Non-Substrate	C21(984); C15(858); C2(767)	Non-Substrate	Non-Substrate	Non-Substrate
**CYP2C19_K_m**	Non-Substrate	Non-Substrate	Non-Substrate	Non-Substrate	61.866	Non-Substrate	Non-Substrate	Non-Substrate
**CYP2C19_V_{max}**	Non-Substrate	Non-Substrate	Non-Substrate	Non-Substrate	2.35	Non-Substrate	Non-Substrate	Non-Substrate
**CYP2C19_C_{Lint}**	Non-Substrate	Non-Substrate	Non-Substrate	Non-Substrate	0.53	Non-Substrate	Non-Substrate	Non-Substrate
**CYP2D6_Inh**	No	Yes	Yes	Yes	No	Yes	Yes	Yes
**CYP2D6_Substr**	No	No	No	No	No	No	No	No
**CYP2D6_Sites**	Non-Substrate	Non-Substrate	Non-Substrate	Non-Substrate	Non-Substrate	Non-Substrate	Non-Substrate	Non-Substrate
**CYP2D6_Km**	Non-Substrate	Non-Substrate	Non-Substrate	Non-Substrate	Non-Substrate	Non-Substrate	Non-Substrate	Non-Substrate
**CYP2D6_Vmax**	Non-Substrate	Non-Substrate	Non-Substrate	Non-Substrate	Non-Substrate	Non-Substrate	Non-Substrate	Non-Substrate
**CYP2D6_CLint**	Non-Substrate	Non-Substrate	Non-Substrate	Non-Substrate	Non-Substrate	Non-Substrate	Non-Substrate	Non-Substrate
**CYP2E1_Substr**	No	No	No	No	No	No	No	No
**CYP2E1_Sites**	Non-Substrate	Non-Substrate	Non-Substrate	Non-Substrate	Non-Substrate	Non-Substrate	Non-Substrate	Non-Substrate
**CYP3A4_Inh**	Yes	No	Yes	No	Yes	No	No	No
**CYP3A4_Substr**	No	No	No	No	No	No	No	Yes
**CYP3A4_Sites**	Non-Substrate	Non-Substrate	Non-Substrate	Non-Substrate	Non-Substrate	Non-Substrate	Non-Substrate	C22(856); C20(846); C9(710);C19(605)
**CYP3A4_Km**	Non-Substrate	Non-Substrate	Non-Substrate	Non-Substrate	Non-Substrate	Non-Substrate	Non-Substrate	46.37
**CYP3A4_Vmax**	Non-Substrate	Non-Substrate	Non-Substrate	Non-Substrate	Non-Substrate	Non-Substrate	Non-Substrate	6.36
**CYP3A4_CLint**	Non-Substrate	Non-Substrate	Non-Substrate	Non-Substrate	Non-Substrate	Non-Substrate	Non-Substrate	15.23
**CYP3A4_HLM_Km**	Non-Substrate	Non-Substrate	Non-Substrate	Non-Substrate	Non-Substrate	Non-Substrate	Non-Substrate	1055.87
**CYP3A4_HLM_V_{max}**	Non-Substrate	Non-Substrate	Non-Substrate	Non-Substrate	Non-Substrate	Non-Substrate	Non-Substrate	1.25
**CYP3A4_HLM_C_{Lint}**	Non-Substrate	Non-Substrate	Non-Substrate	Non-Substrate	Non-Substrate	Non-Substrate	Non-Substrate	1.19
**CYP_HLM_C_{Lint}**	19.5	12.63	17.30	13.97	17.58	7.72	7.3	20.34
**CYP3A4_Inh_midaz**	Yes	Yes	Yes	Yes	Yes	Yes	Yes	Yes
**CYP3A4_Inh_testo**	No	No	No	No	No	No	No	No
**CYP3A4_Ki_midaz**	63.13	14.75	28.07	14.3	28.42	14.89	14.64	16.87
**CYP3A4_Ki_testo**	4.36	2.33	4.24	3.89	3.20	1.9	3.01	8.18
**UGT1A1**	Yes	Yes	Yes	Yes	Yes	Yes	Yes	Yes
**UGT1A3**	Yes	Yes	Yes	Yes	Yes	Yes	Yes	Yes
**UGT1A4**	No	No	No	No	No	No	No	Yes
**UGT1A6**	No	No	No	No	No	No	No	No
**UGT1A8**	Yes	Yes	Yes	Yes	Yes	Yes	Yes	No
**UGT1A9**	Yes	Yes	Yes	Yes	Yes	Yes	Yes	Yes
**UGT1A10**	Yes	Yes	Yes	Yes	Yes	Yes	Yes	Yes

**UGT2B7**	Yes	No	No	No	No	No	No	No
**UGT2B15**	Yes	Yes	Yes	Yes	Yes	Yes	Yes	Yes

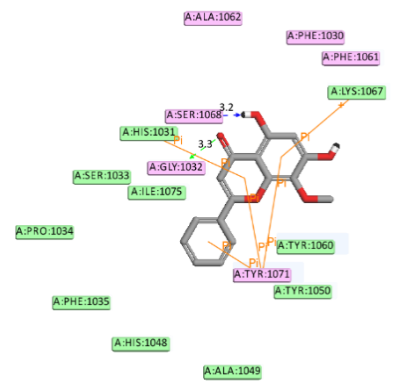

Compound F2

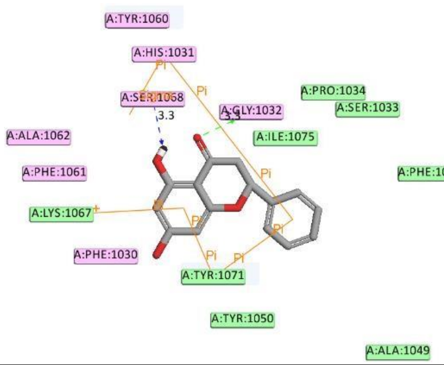

Compound F3

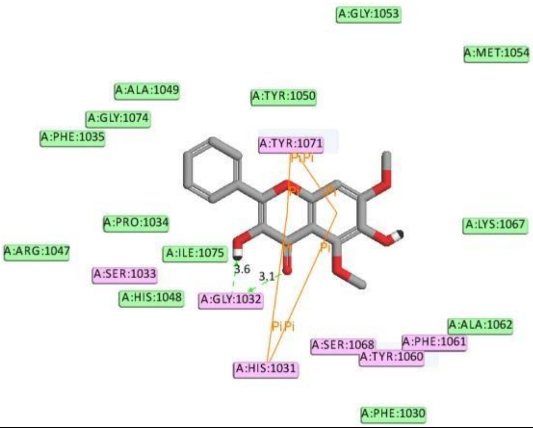

Compound F8

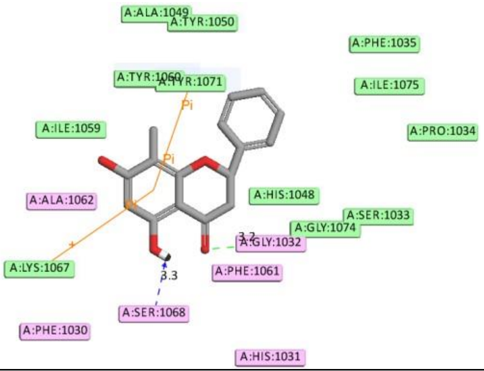

Compound F11

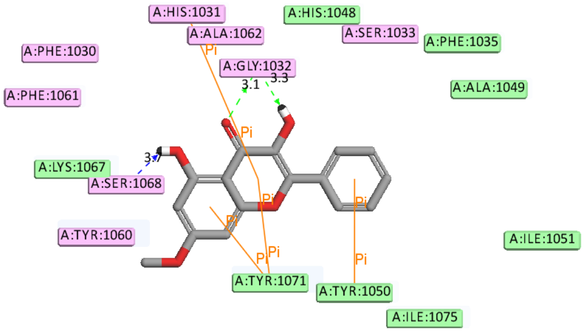

Compound F13

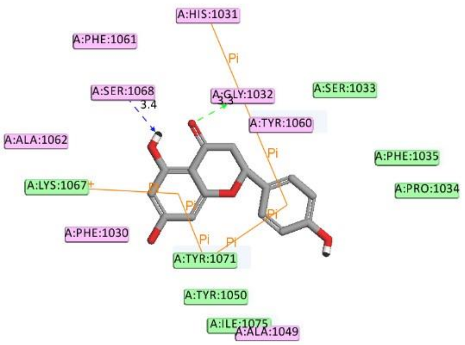

Compound F20

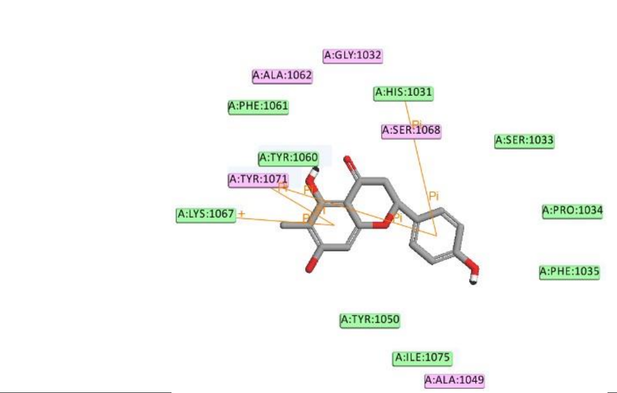	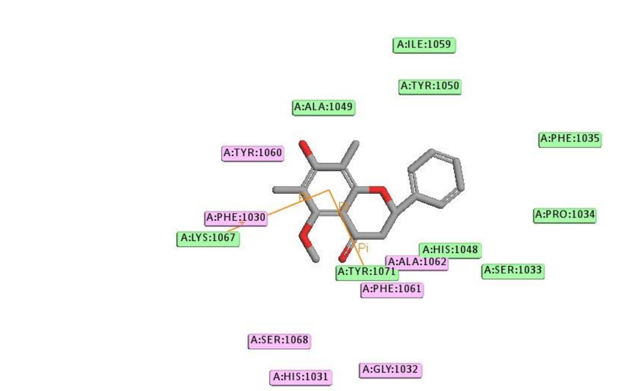
Compound F21	Compound F25

Figure S1: Two-dimensional diagrams illustrating protein-ligand interactions between candidate compounds and target receptor. Broken arrow represents the hydrogen bonding, and orange line represents the pi-interactions with length in Å.

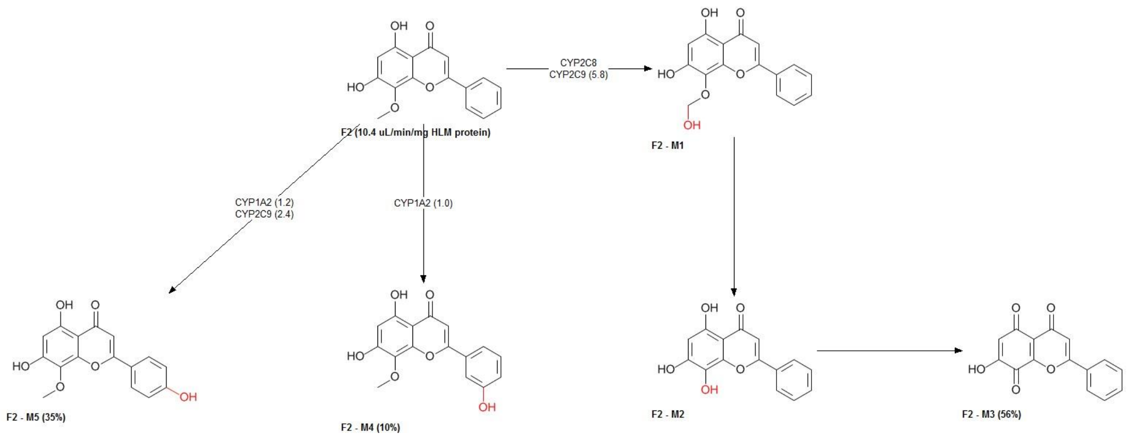

Figure S4.6.2a: The predictive metabolites and sites of metabolism of candidate compound F2**.**

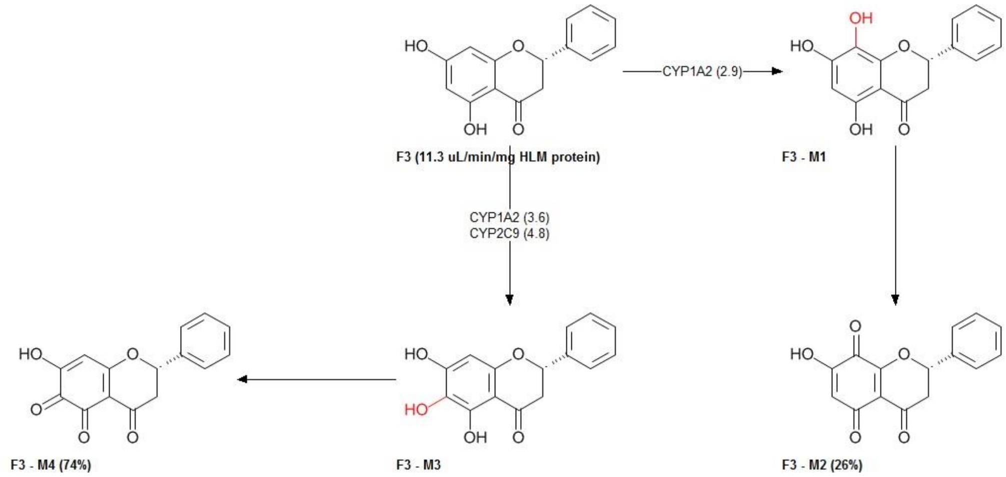

Figure S4.6.2b: The predictive metabolites and sites of metabolism of candidate compound F3.

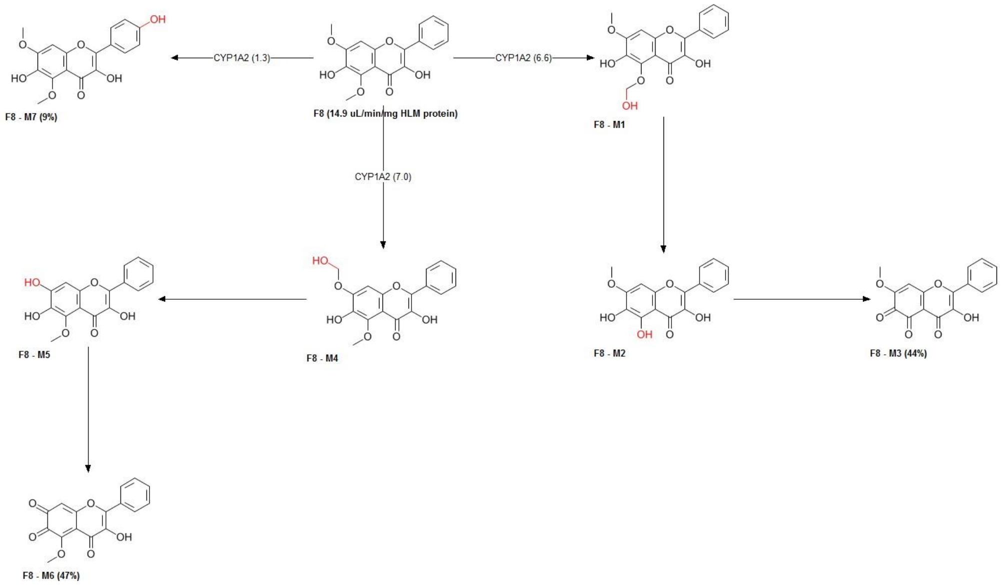

Figure S2c: The predictive metabolites and sites of metabolism of candidate compound F8.

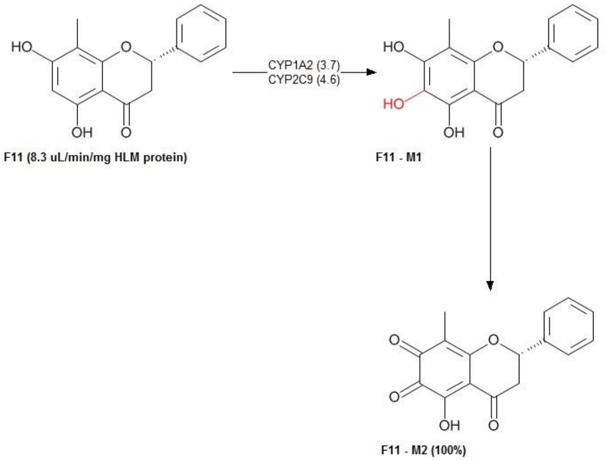

Figure Sd: The predictive metabolites and sites of metabolism of candidate compound F11.

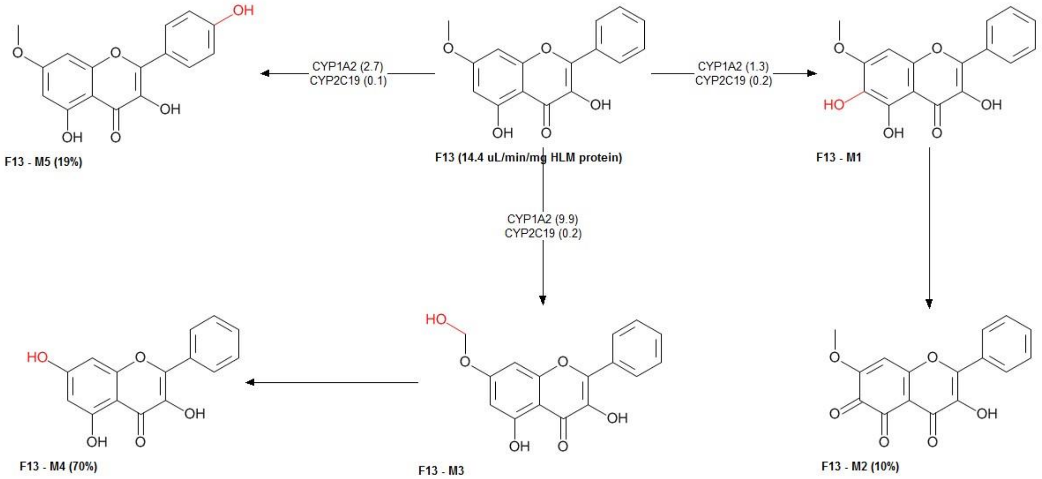

Figure S2e: The predictive metabolites and sites of metabolism of candidate compound F13.

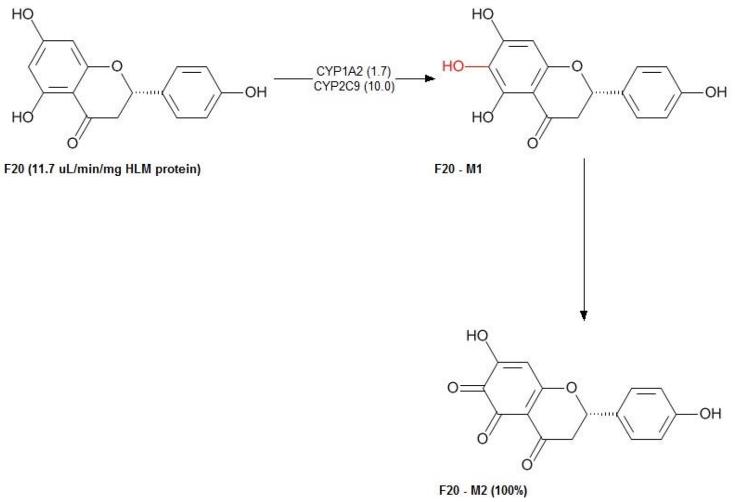

Figure S2f: The predictive metabolites and sites of metabolism of candidate compound F20.

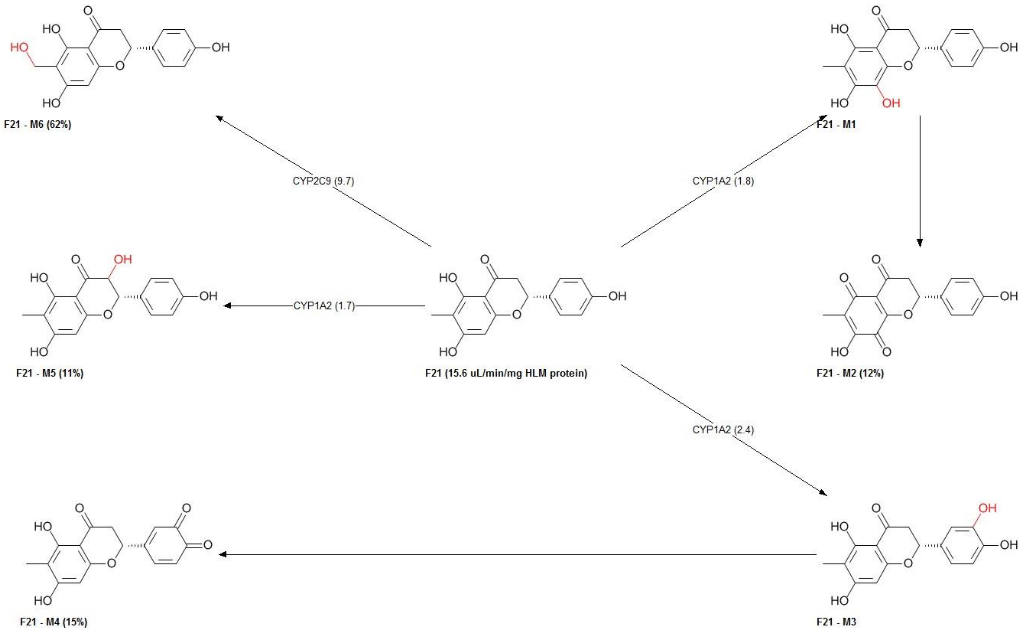

Figure S2g: The predictive metabolites and sites of metabolism of candidate compound F21**.**

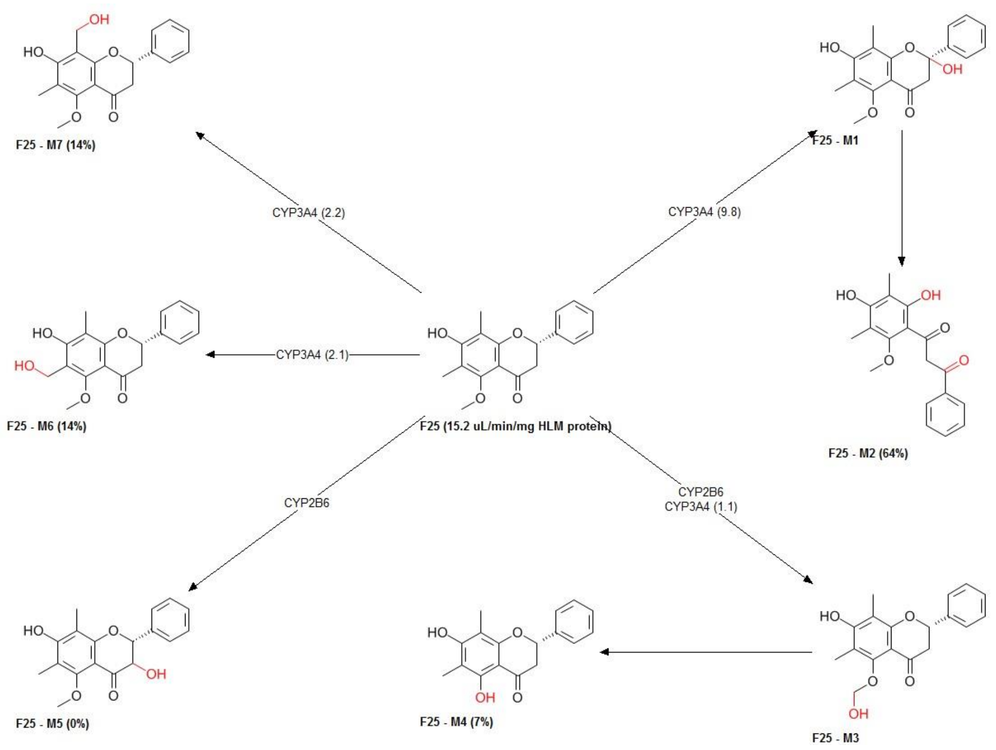

Figure S2h: The predictive metabolites and sites of metabolism of candidate compound F25.
